# MtDNA genetic diversity and phylogeographic insights into giant domestic pigeon (*Columba livia domestica*) breeds: connections between Central Europe and the Middle East

**DOI:** 10.1016/j.psj.2024.104310

**Published:** 2024-09-07

**Authors:** K. Balog, A.S. Wadday, B.A. Al-Hasan, G. Wanjala, Sz. Kusza, P. Fehér, V. Stéger, Z. Bagi

**Affiliations:** ⁎ Centre for Agricultural Genomics and Biotechnology, University of Debrecen, Debrecen, Hungary; † Doctoral School of Animal Science, University of Debrecen, Debrecen, Hungary; ‡ Faculty of Agricultural and Food Sciences and Environmental Management, University of Debrecen, 4032, Debrecen, Hungary; § Department of Medical Laboratory Technology, College of Medical Technology, The Islamic University, Najaf, Iraq; # Department of Laboratory, Al-Najaf Veterinary Hospital, Najaf, Iraq; ¶ Department of Veterinary Microbiology, College of Veterinary Medicine, University of Al-Qadisiyah, Diwaniyah City, Iraq; ǁ Institute of Animal Sciences and Wildlife Management, University of Szeged, Hungary; ⁎⁎ Department of Genetics and Genomics, Institute of Genetics and Biotechnology, Hungarian University of Agriculture and Life Sciences, Gödöllő, Hungary

**Keywords:** COI region, *Columba livia domestica*, genetic diversity, mtDNA, squab pigeon

## Abstract

Humans have selectively bred domestic pigeons (*Columba livia domestica*) to create breeds with a diversity of shapes, colors and other attributes. Since Darwin, the domestic pigeon has always been a popular model species for scientific research because of its richness of form, colouration and behaviour. It is believed that the world's squab pigeon industry uses breeds and hybrids from the Mediterranean region. An exception is the indigenous giant pigeon breeds of the Carpathian Basin, whose origin is not known. Therefore, our aims were 1) to understand the phylogenetic relationships of giant pigeons, which sheds light on the origin of Hungarian breeds and their relationship to the Mediterranean giant pigeon breed group; 2) to contribute molecular genetic data to the genealogy of 2 Iraqi pigeon breeds close to the pigeon domestication center, including the culturally important Iraqi Red Pigeon, and 3) to compare the genetic diversity of European and Middle Eastern domestic pigeon populations and to draw conclusions on the phylogenetic relationships between pigeon breeds and molecular clues to their different breeding practices of both regions. A 655-bp-long sequence of the cytochrome oxidase 1 (**COI**) region of the mitochondrial DNA was studied in a total of 276 pigeons (19 breeds). A total of 27 haplotypes were found, of which 22 were unique. The highest genetic diversity was found in the Carpathian Basin, and the lowest in the Iraqi region. STRUCTURE analysis revealed low structurality, K=3 was the most likely. The majority of the samples belong to the most ancient haplotype H_2=219, however the Jacobin pigeon is on a very separate evolutionary branch with a large number of mutations. None of the 19 breeds investigated in this study have been previously studied in phylogenetics, and most of these breeds have potential as squab pigeons, and have good meat forms for utilization, therefore the results of this study may also be of help to the squab pigeon industry.

## INTRODUCTION

The domestic pigeon (*Columba livia domestica*) is still very popular as a food source from North America to Europe, and from the Middle East to China due to the relatively low investment, low feed and keeping costs, and the high biological value of its meat (Asaduzzaman et al., 2012). Therefore, one of the most significant utilization directions – in addition to breeding as a hobby – is the use in meat production, which provides food staples both for the poorest and for the consumers of premium products. Despite its popularity, the original geographic distribution of the species is difficult to determine due to its long history and intensive domestication process (Darwin, 1859; Gibbs et al., 2001; Holmes and Ottinger, 2006; Ashley et al., 2011; Shapiro et al., 2013). However, the scarce descriptions available suggest that domestic pigeons have spread rapidly over the years from Central Asia, through pre-Asia, to Assyria and then Syria (Gibbs et al., 2001; Driscoll et al., 2009; Phillips, 2009; Shapiro et al., 2013). Subsequently, under the influence of environment and culture, they diversified along with humans throughout Eurasia (Pacheco et al., 2020). The crossbreeding of giant breeds originated from the Mediterranean region. Later, the squab breeds were developed mainly in America and Europe (Mead, 2004); large-scale production began in America at the beginning of the 20th century, and in Europe in the 1960s (Jilly, 2018). For the sustainability of the squab sector and the exploitation of genetic reserves understanding the phylogenetics of this species, especially the economically important breeds with potential for large-scale meat production, is important (Gilbert and Shapiro, 2014; Jilly, 2018).

In Europe, the Carpathian Basin is a biodiversity hotspot due to its geographic characteristics and it is plays a role in the spread of many wild and domestic species (Kajtoch et al., 2016; Mráz and Michał, 2016). Thus, Hungary, located in the middle of the basin, is a promising area for studying the domestication history of the domestic pigeon. In the past, historical and geographical factors also shaped the situation of Hungarian pigeon breeding, as several trade routes crossed historical Hungary (Bagi and Kusza, 2014). Therefore, the ancestors of today's Hungarian breeds probably came partly from the east and partly from the west (Gál, 2007; Kiss and Béres, 2008). Because of the former Ottoman Empire, which covered large areas of both modern-day Iraq and present-day Hungary, several European pigeon breeds probably originated from the Middle East, which was the initial center of their domestication, based on the historical account of domesticating the domestic pigeon. Furthermore, several European pigeon breeds have successfully reached Iraq, providing further evidence of mutual links.

Despite the opportunities in the squab pigeon sector, the domestic pigeon is still one of the less researched domestic animal species, both from a biotechnological and a molecular genetic point of view (Stringham et al., 2012; Shapiro et al., 2013; Domyan et al., 2016; Pacheco et al., 2020). Given the limited number of phylogenetic studies undertaken on domestic pigeon breeds so far, it is crucial to prioritize the investigation of their phylogenetic relationships and identification using mitochondrial DNA in extensive exploratory research (Gao et al., 2016; Kowalczyk et al., 2021; Salem et al., 2022; Raza et al., 2023).

Previously, various genetic methods have been successfully used to explore genetic diversity and phylogenetic relationships, which has been effective in expanding our poor knowledge of the breeds (Ramadan et al, 2011; Casanova, 2013; Firyal et al., 2014; Rafiq et al., 2020). Latest studies have been carried out to understand evolutionary processes among wild and domesticated pigeon populations. For example, in 2020 Pacheco et al. generated genome-wide data on nearly 200 individuals using the genotyping sequence (**GBS**) method in order to expand the understanding of the complex evolutionary relationships between pigeon breeds. Hou et al. (2021) re-sequenced 7 pigeon breeds with full genome sequencing, including commercial breeds, ornamental breeds and local breeds. However, in sparsely studied geographical areas and for large sample sizes the mitochondrial marker and the COI region itself can be an effective tool in their own right at the current stage of phylogenetic studies of domestic pigeon breeds (Nabholz et al., 2009; Saif et al., 2012; Khan and Arif, 2013). Furthermore, the latest technologies are very efficient (Bello et al., 2023), but not cost-effective. Also, the relevant avian literature reveals that there are relatively few cases where nuclear markers conflict with mitochondrial markers in such a way that they are incompatible (Edwards et al., 2005; Zink and Barrowclough, 2008; Allio et al., 2017).

The distinction between pigeon breeds and breed groups, as well as whether this distinction is biologically relevant, is a very controversial issue nowadays (Stringham et al., 2012; Shapiro et al., 2013).

In this study we aim 1) to understand the phylogenetic relationships of giant pigeons, which sheds light on the origin of Hungarian breeds and their relationship to the Mediterranean giant pigeon breed group;

2) to contribute molecular genetic data to the genealogy of two Iraqi pigeon breeds which evolved close to the pigeon domestication centre, including the culturally important Iraqi Red Pigeon, and

3) to compare the genetic diversity of Central European and Middle Eastern domestic pigeon populations and to draw conclusions on the phylogenetic relationships between pigeon breeds and on molecular clues to the different breeding practices of both regions.

## MATERIALS AND METHODS

### Sampling

Nineteen pigeon breeds are involved in this study (Table 1). The detailed historical origin and phenotypic description of the breeds examined in this study are summarized in Supplementary File 1. In selecting the giant breeds, we considered official breed standards and defined them as breeds larger than the average size of a typical pigeon (e.g., racing pigeon), which typically weigh between 400-500 grams and measure about 28-38 centimeters in length. Breeds with average adult weights exceeding 700 grams were classified as "giant" in this study.

**Table 1:** Number of samples studied and grouping types.

Breeds	Sample size (n)	Traditional breed group	Origin of the breeds	Type of sample
**Bokhara Trumpeter**	7	Trumpeter pigeons	Asian Breeds	blood
**Blue Sovater**	11	Utility pigeons	Hungarian Squab Pigeons	blood
**Buga pigeon**	12	Utility pigeons	Breeds from the Hungarian Great Plain	blood
**Carnao**	10	Utility pigeons	Mediterranean Pigeons	blood
**Hubbel**	10	Squab pigeons	American Breeds	blood
**Hungarian Chicken pigeon**	8	Poule Type pigeons	Hungarian Squab Pigeons	blood
**Hungarian Cropper**	14	Cropper pigeons	Breeds from the Hungarian Great Plain	blood
**Hungarian Domestic pigeon**	9	Utility pigeons	Breeds from the Hungarian Great Plain	blood
**Hungarian Giant pigeon**	15	Utility pigeons	Breeds from the Hungarian Great Plain	blood
**Hungarian Peasant pigeon**	10	Utility pigeons	Breeds from the Hungarian Great Plain	blood
**Iraqi Raabi pigeon**	50	Utility pigeons	Asian Breeds	FTA card
**Iraqi Red pigeon**	47	Tumbler pigeons	Asian Breeds	FTA card
**Jacobin**	10	Structure pigeons	Asian Breeds	blood
**King**	11	Poule Type pigeons	American Breeds	blood
**Mirthys**	10	Squab pigeons	Mediterranean Pigeons	blood
**Mondain**	10	Utility pigeons	Mediterranean Pigeons	blood
**Salonta Giant**	13	Utility pigeons	Breeds fromt the Hungarian Great Plain	blood
**Runt pigeon**	10	Utility pigeons	Mediterranean Pigeons	blood
**Texan**	9	Utility pigeons	American Breeds	blood

We utilized several different theoretical grouping principles, which were as follows: 1) According to traditional breed groups, which classifies the breeds studied into 7 groups (Supplementary File 1, Supplementary File 2).

(2) According to the geographical area of origin (origin of the breeds). This grouping is based on the original evolution sites of the breeds, and we distinguish 5 groups (Supplementary File 1, Supplementary File 2). We created two subgroups within the Carpathian Basin, because one of them contains breeds belonging to a supposedly ancient lineage, which are also phenotypically similar to each other (eg. giant, giant). There is another morphologically distinct subgroup, a squab group, which have different body proportions and breed characteristics and clearly belongs to a different lineage.

Total blood samples were collected from flocks of pigeon breeders living in Hungary and Romania between 2018 and 2023. Sampling locations are summarized in Supplementary File 2. The blood samples of the Iraqi breeds were also collected in EDTA-containing blood tubes and stored at -20°C until transferring 100 µl to FTA cards (Qiagen, Germany), which were then stored in a dry place at room temperature.

The study was approved and carried out in accordance with the local ethics committee's Guidelines of the University of Debrecen under the registration number 20/2023/DEMÁB.

### DNA Isolation

DNA isolation from the blood samples was performed at the Centre for Agricultural Genomics and Biotechnology, University of Debrecen. The protocol by Zsolnai and Orbán (1999) was used for the 179 blood samples, and the MagCore (for dried blood spot) Genomic DNA Tissue Kit (RBC Bioscience Corp., Taiwan) was used for isolating DNA from 97 FTA cards, using the MagCore Automated Nucleic Acid Extractor robot (RBC Bioscience Corp., Taiwan) (Table 1) at the Institute of Genetics and Biotechnology, Hungarian University of Agriculture and Life Sciences. The concentration of isolated DNA was measured using a NanoDrop1000 spectrophotometer (ThermoFisher Scientific, USA).

### PCR

Three primer pairs were designed to amplify the COI region using the Primer3 software, (Koressaar et al., 2018) and the domestic pigeon (*Columba livia domestica*) mitochondrion, complete genome (Genbank ID: NC_013978.1) found in the NCBI Gene Bank. The characteristics of the primers used in the PCR reactions are shown in Table 2.

**Table 2:** Characteristics of the primers used during PCR analysis.

Primer	Forward primer sequence (5′-3′)	Reverse primer sequence (5′-3′)	Amplified product length (bp)	Adhesion temperature (°C)
**G_728**	CTGCTCACAGACCGAAACCT	GACGCCTACACCCTGTGAAA	728	64
**G_901**	TAGCCTCCTCCACAGTCGAA	TGGGCTTTAGCTCATGTGGG	901	64
**G_1299**	CTGCTCACAGACCGAAACCT	TGATGGAAGGGCGAGTAGGA	1299	64

Samples were amplified using PCR (Singh and Kumar, 2001;Vinay and Kumar, 2001) in the following reaction mixture at a total volume of 50 µL: 18 µl dH2O, 5 µL dNTP (2 mM), 10 µL 5x Colorless GoTaq Flexi Buffer 1.2 mM, 8 µl MgCl_2_ (25 mM), 1 µL 1 pmol/µl forward primer 25 nmol, 1 µl 1 pmol/µl reverse primer 25 nmol, 1 µl DreamTaq Flexi DNA Polymerase 1,000 unit, 6 µl isolated gDNA. PCR conditions: 94°C 4:00 min, 94°C 1:00 min, 64°C 1:00 min, 72°C 1:00 min, 72°C 10:00 min, 10°C ∞. Amplification of the sequence defined by the primer pair was performed using the Biometra Tone PCR machine (Analytik Jena GmbH+Co. Germany). After PCR, the success of amplification was verified by agarose gel (2%) electrophoresis. Sequencing was carried out as a service by the Macrogen Europe laboratory in Amsterdam.

### Data Analysis

Electropherograms were checked by eye, and the sequences were aligned using ClustalW (Larkin et al., 2007). To shorten the sequences and draw the dendrogram, we used MEGA11 (Tamura et al., 2021) software. DnaSP 6 software (Rozas et al., 2017) was used to measure the number and distribution of haplotypes, the number of polymorphisms and the nucleotide frequency values. Arlequin 3.5.2.2 software (Excoffier and Lischer, 2010) was used to perform fixation index (Fst), genetic distance and AMOVA tests. The phylogenetic tree was constructed using Neighbor-Joining analysis to illustrate evolutionary relationships with Network 5.0.1.1 (Rozas et al., 2017) software package. Eurasian Collared Dove (*Streptopelia decaocto*) was used as an outgroup in the analysis of haplotype relationships with the Tamura-Nei method (accession number on NCBI: MF381976.1). The haplotypes detected in this study are available in the NCBI genebank database under the accession number PP952127-PP952153. To evaluate patterns of genetic structure and to create clusters in the sample set, the Structure 2.3.4. version (Pritchard et al., 2010) was used. The set parameters were as follows: MCMC algorithm in 100,000 repetitions, 10,000 burn-in steps and 10 iterations per different K value (K=number of groups). The results of the analysis were validated using the STRUCTURE Harvester (Earl and vonHoldt, 2011) program, in which the Evanno method (Evanno et al., 2005) was applied to determine the most probable grouping. Based on the genetic distance values, in order to visualize the results at the level of individuals, principal coordinate analysis (PCoA) was used with the Genalex Excel extension (Peakall and Smouse 2006, 2012).

## RESULTS

### Genetic Diversity Indices

The results are presented starting with indices of genetic diversity. Genetic diversity indices based on the traditional breed grouping are summarized in Table 3. Structure pigeons had the highest number of polymorphisms (120), followed by Utility pigeons (111) and Squab pigeons (35). The lowest number of polymorphisms were found in Poule-type pigeons (1) and Trumpeter pigeons (1). In proportion to the number of polymorphisms, the Structure pigeons (H_d_=0.778 ± 0.137) and the Utility pigeons (H_d_ = 0.396 ± 0.049) groups have the highest values of haplotype diversity, and the Squab pigeon group also showed a high value (H_d_ = 0.363 ± 0.131). The lowest haplotype diversity values were found in the Poule Type pigeons (H_d_=0.105 ± 0.092) and the Tumbler pigeons (H_d_ = 0.125 ± 0.065) groups.

**Table 3:** Diversity indices based on the traditional breed groups.

Indices Grouping	Number of elements (n)	Number of polymorphisms	Number of haplotypes	Haplotype diversity (H_d_) ± SD	Nucleotide diversity (p) ± SD
**Cropper pigeons**	14	2	3	0.275 ± 0.148	0.001± 0.002
**Poule Type pigeons**	19	1	2	0.105 ± 0.092	0.001± 0.001
**Structure pigeons**	10	120	6	0.778 ± 0.137	0.189± 0.102
**Squab pigeons**	20	35	4	0.363 ± 0.131	0.017± 0.010
**Trumpeter pigeons**	7	1	2	0.286 ± 0.196	0.001± 0.002
**Tumbler pigeons**	47	4	4	0.125 ± 0.065	0.001±0.001
**Utility pigeons**	159	111	17	0.396 ± 0.049	0.015±0.009

Nucleotide diversity was generally low, except for Structure pigeons (π = 0.189 ± 0.102), Squab pigeons (π = 0.017 ± 0.010), and Utility pigeons (π = 0.015 ± 0.009).

Table 4 presents the genetic diversity indices of pigeons grouped according to their origin. The Table shows that the number of polymorphisms is high in the Asian breeds (129) and the Hungarian breeds (91). Therefore, the number of haplotypes is also high in these two groups: Asian group (15), Breeds from the Great Hungarian Plain (13); however, other groups had low haplotype numbers. The Mediterranean pigeon's group (H_d_ = 0.576 ± 0.078) had the highest haplotype diversity. High haplotype diversities were also detected in the Breeds from the Great Hungarian Plain (H_d_ = 0.486 ± 0.071) and the Asian breeds (H_d_ = 0.305 ± 0.057) groups. Similar results were obtained for nucleotide diversity values: Asian breeds (π = 0.031 ± 0.017), Breeds from the Great Hungarian Plain (π = 0.025 ± 0.013) and Mediterranean pigeons (π=0.018 ± 0.010) have higher values. Supplementary File 3 presents the diversity indices calculated by breed groups.

**Table 4:** Diversity indices based on the origin of the breeds.

Indices Group	Number of elements (n)	Number of polymorphisms	Number of haplotypes	Haplotype diversity (H_d_) ± SD	Nucleotide diversity (π) ± SD
**American pigeons**	30	2	3	0.191 ± 0.093	0.001± 0.001
**Asian pigeons**	114	129	15	0.305 ± 0.057	0.031± 0.017
**Breeds from the Great Hungarian Plain**	73	91	13	0.486 ± 0.071	0.025± 0.013
**Hungarian Squab pigeons**	19	18	2	0.105 ± 0.092	0.009 ± 0.006
**Mediterranean pigeons**	40	65	6	0.576 ± 0.078	0.018± 0.010

Higher values for haplotype diversity were detected in Jacobin (H_d_=0.778 ± 0.137), Salonta Giant (H_d_=0.731 ± 0.096), Hungarian Giant (H_d_=0.629 ± 0.125), and Mondain (H_d_=0.533 ± 0.180) pigeons, whereas Hungarian Chicken and Texan (0.000) pigeons exhibited no haplotype diversity (0.000) possibly due to the low number of samples in these breeds (Hungarian Chicken pigeon n = 8, Texan n = 9). The Iraqi Red and Raabi pigeons showed comparable values, but the number of polymorphisms is higher in the Iraqi Red (4) than in the Iraqi Raabi pigeon (2). There was an insignificant difference between Iraki Red (H_d_=0.125 ± 0.065) and Raabi (H_d_ = 0.191 ± 0.074), which also clearly shows the uniqueness and isolation of these two breeds.

### Genetic Distance Analysis

The **Fst** value (Fixation index) represents the proportion of nucleotide sites where sequences contain different bases in the pairs compared. If Fst<0.01, the 2 given sets of samples are considered close (Priskin, 2010). Table 5 summarizes the values of the genetic distance of the traditional breed grouping based on the Fst values per pairwise comparison.

**Table 5:** Pairwise Fst comparison between the traditional breed (the asterisk marks the statistically significant values (*p* < 0.05), the values marked with green represent high values, and the low values are marked with red).

Overall, low Fst values were observed across all groups, although the *a priori* populations exhibited different genetic distances. Table 5 shows that the Structure group has a high genetic distance in all comparisons. Negative Fst values indicate that the relationship between the two compared groups is very close, with an almost negligible genetic distance. It is also possible that the number of samples in the groups may be insufficient for accurate testing (Gerlach et al., 2010; Priskin, 2010). The Fst values indicating genetic distances between groups based on breed origin are summarized in Supplementary File 4, while those based on breed type are summarized in Supplementary File 5. The values in Supplementary File 4 are not statistically significant and are quite small. The greatest distance is between the American and Hungarian Squab pigeon groups (0.022), whereas Mediterranean pigeons are relatively close to the Asian (0.008) and Hungarian Squab breeds (0.007). Breeds from the Great Hungarian Plain group show negative Fst values compared to both Mediterranean and Hungarian Squab breeds, suggesting that there is more variation within the groups than between them. This indicates a small genetic distance, reflecting the close relatedness of giant pigeons regardless of their region of origin.

The genetic relationships between the breeds studied are shown through the comparisons presented in Supplementary File 5. Interestingly, the Hungarian Giant and the Hungarian Cropper (0.002) are found to be closely related, despite belonging to different breed groups. One Hungarian breed is the Blue Sovater, which originated from Hungary and is close to all other breeds except the Jacobin (0.470), the Iraqi Red (0.126) and the Iraqi Raabi (0.149). Each value is statistically significant (p<0.05), possibly because the breed is very young and has not yet developed its unique genetic character. The Hungarian Domestic pigeon shown the closest relationship with the Buga pigeon (0.004).

### Analysis of Molecular Variance

Table 6 summarizes the results of the AMOVA analysis according to the groupings used in this study. The grouping by breed revealed that a larger proportion (69.48%) of the genetic variation occurs within populations, but the value of variation among the populations was 30.52%, which is also significant and demonstrates the individual character of the breeds.

**Table 6:** The result of the AMOVA test for groups.

	Source of variation	df	Sum of squares	Variance components	Percentage of variation (%)
**Based on the traditional breed groups**	**Among populations**	6	191.143	0.92804	35.07
	**Within populations**	269	462.231	1.71833	64.93
**Based on the studied breeds**	**Among populations**	18	217.926	0.74415	30.52
	**Within populations**	257	435.447	1.69435	69.48

### Principal Coordinate Analysis

**Figure 1:**
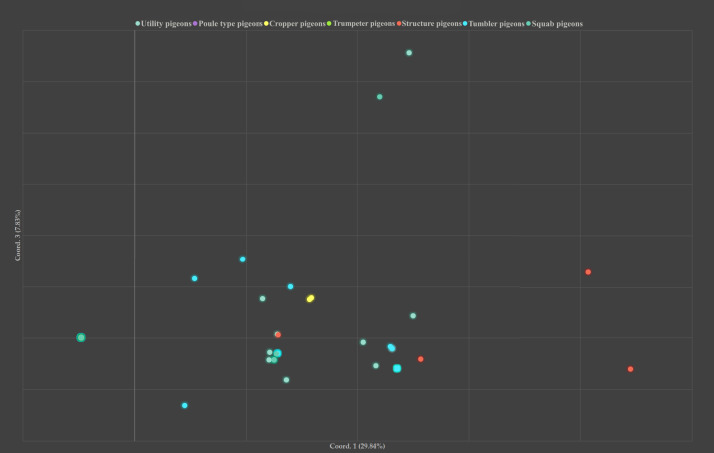
Principal coordinate analysis (1 vs. 3) based on the traditional breed groups.

Principal coordinate analysis (**PCoA**) was performed on the groups established using the genetic distance values. Figure 1 shows the results by traditional breed grouping, in this figure it was not possible to visualize either the Poule Type pigeon group or the Trumpeter pigeon group. In Supplementary File 6, of the comparison by breed it shown that the differences are more likely to appear on the level of individuals. Supplementary File 7 shows the results based on the grouping according to the origin of the breeds. This Figure does not show a clear pattern, there is a high degree of mixing between individuals.

### Haplotype Relationships Among Breeds and Groups

**Figure 2:**
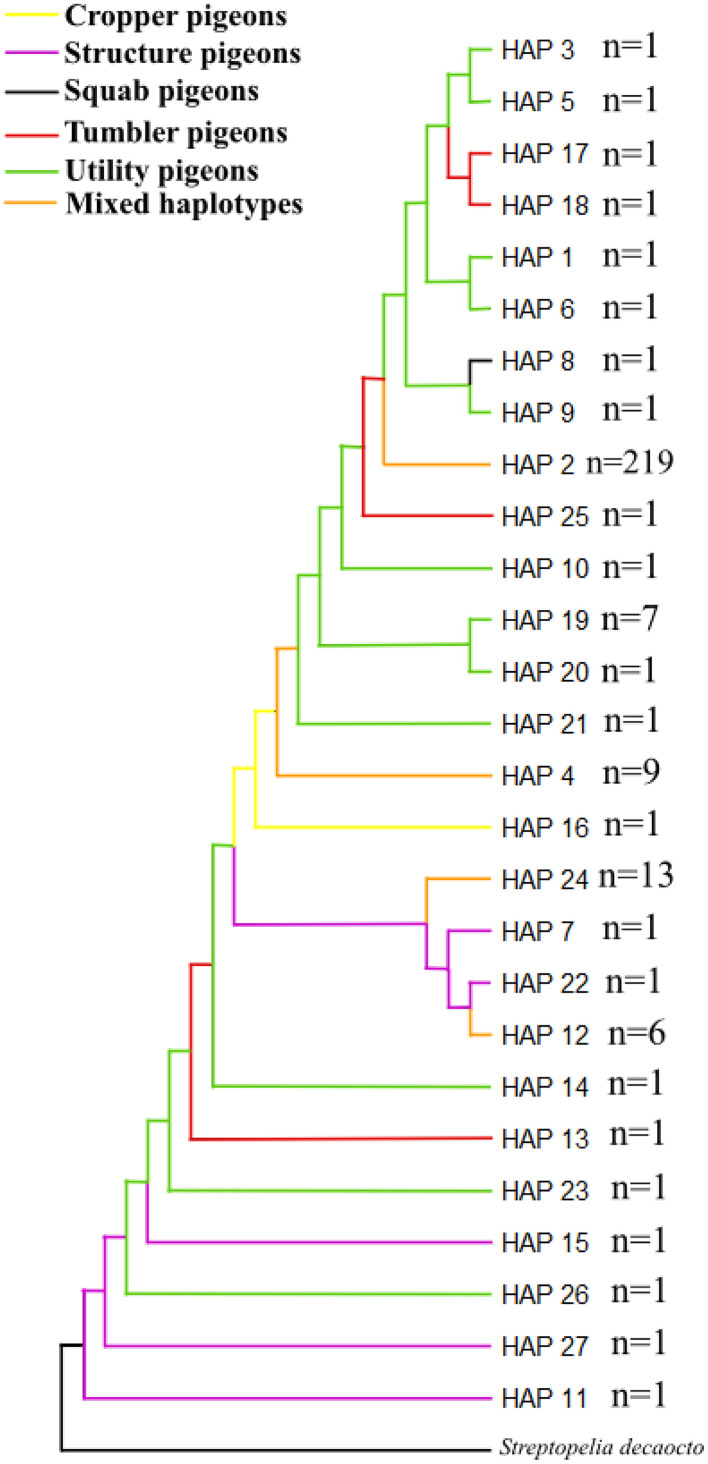
Tamura-Nei dendrogram of haplotype relationships (MEGA 11) based on the origin of the breeds.

Figure 2 shows the relationships among the haplotypes by the grouping based on traditional breed groups. From the 27 haplotypes, the groups share 4 haplotypes (mixed haplotypes marked with orange). The dendrograms for the origin of the breeds are summarized in Supplementary File 8. Haplotype 2 occurs in 219 individuals, mixed from the 4 groups, from which it can be concluded that this haplotype can be considered general and is the most ancient one.

**Figure 3:**
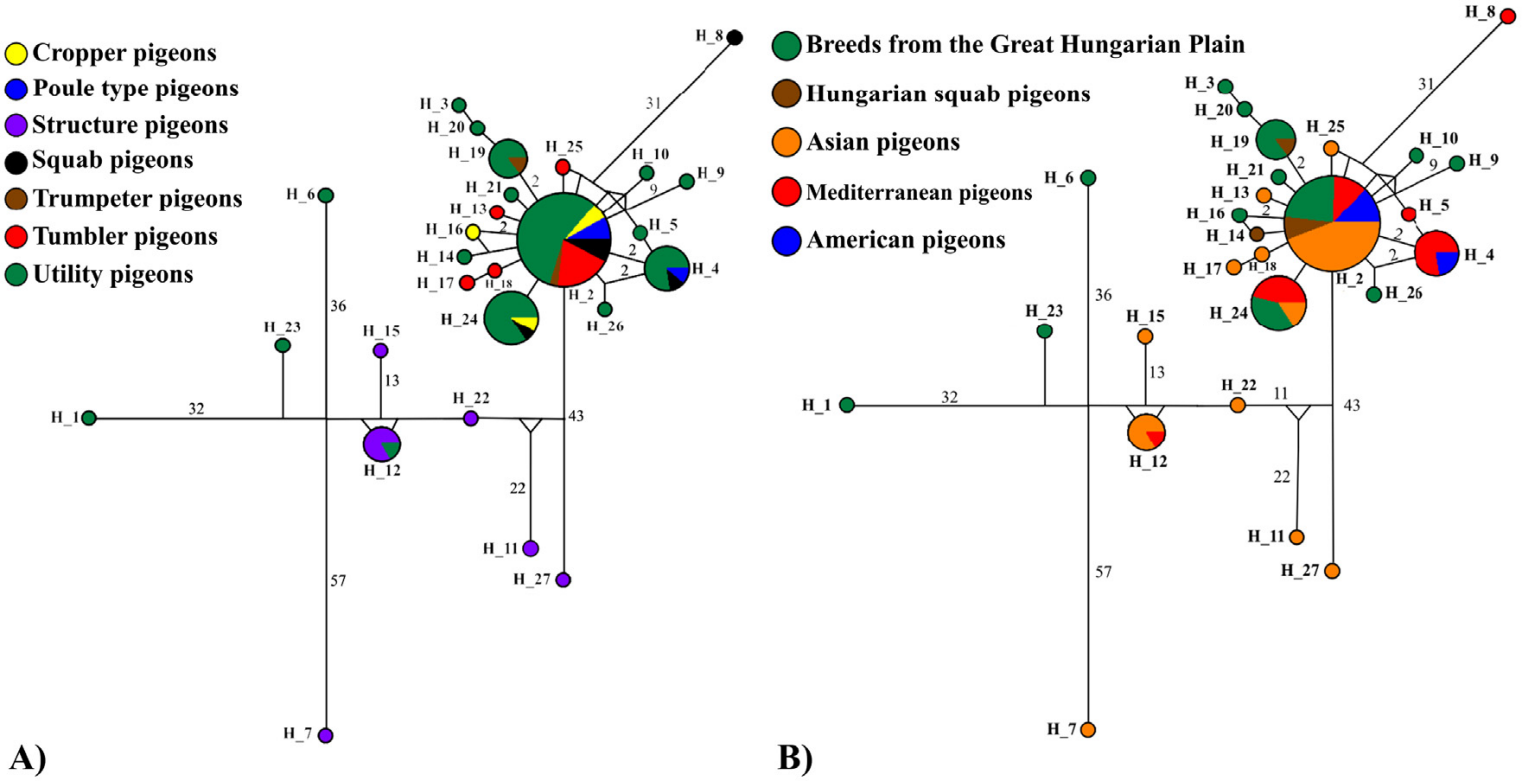
Neighbor-Joining Network diagram of haplotype relationships (A) based on the traditional breed groups, and (B) based on the origin of the breeds.

The distribution of haplotypes was examined by Neighbor-Joining Network analysis according to the two groupings (Figure 3). In the network Figure (Figure 3A), it can be seen that the Utility pigeon group has the most unique haplotypes, and that this group shows great mixing with the other breed groups. The Structure pigeon group is on a separate branch with unique haplotypes from some other Utility pigeon groups. The Cropper pigeon group has only one unique haplotype, and the same is the case with the Squab pigeon group.

Figure 3B shows the haplotype distribution among the examined individuals according to the origin of the breeds. It can be seen that the breeds of the Carpathian Basin are very diverse despite the equalized sample numbers and have many local haplotypes.

There is also mixing with the Mediterranean region in more populous haplotypes, but the Asian connection with the Carpathian Basin is more significant than the Mediterranean direction.

Furthermore, it is shown in Figure 3B that the Mediterranean region always shares a common haplotype with the American region.

In Figure 3, Figure 3, the most frequent and most ancient haplotype appears most clearly, in a star-like arrangement. The Asian breeds, including the Iraqi pigeons, are mostly represented in the central part of the Figure 3B, which reflects the proximity of the Iraqi breeds to the center of domestication. There are some individuals in the Iraqi breeds that do not share the most ancient haplotypes, but these are mostly only one mutation removed from them.

### Cluster Analysis

**Figure 4:**
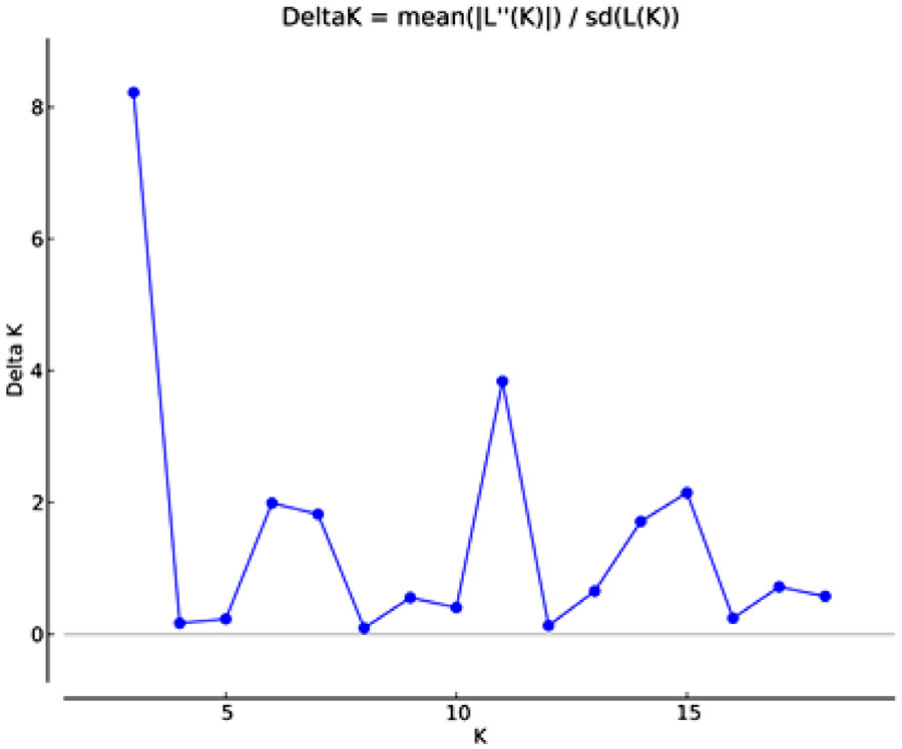
DeltaK value from structure Harvester.

The structure of the populations and the most likely groups they form were determined (using an admixture model) by the STRUCTURE software, which compares the allelic patterns of individuals pairwise and classifies genotypes into different groups without making assumptions. Comparing all the *a priori* populations (K: 1-19), Bayesian analysis revealed that the largest delta K value was for K=3 and then the second peak at K=11 (Figure 4).

**Figure 5:**
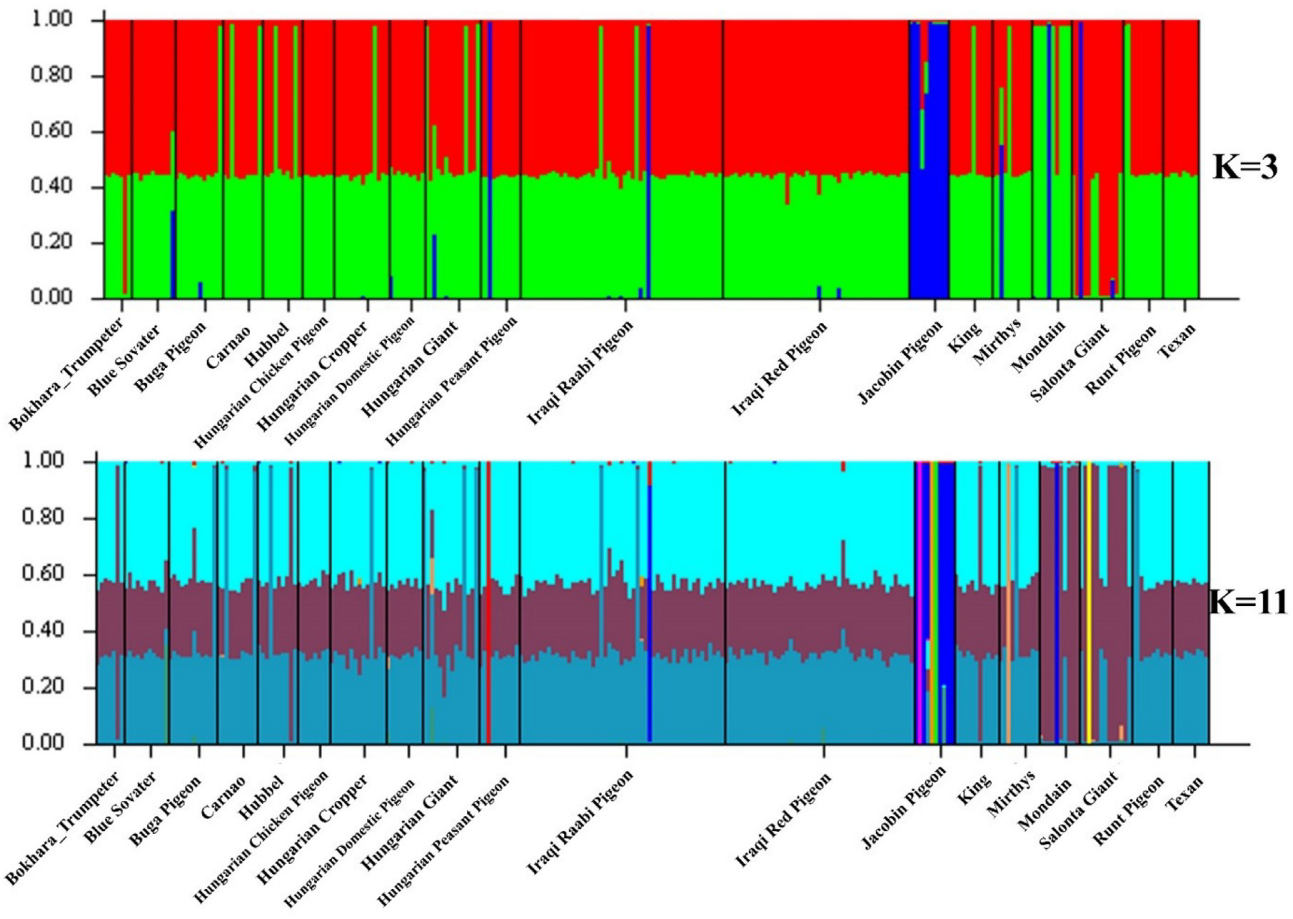
The result of the cluster analysis with STRUCTURE 2.3.4.

Figure 5 shows that there is a high degree of admixture in most populations, but at K=3, 3 populations with more consistent characters can be identified from the analysis. These are not completely pure and uniform, but they have a genetic basis that can be used for distinguishing them. In the case of K=11, however, it can be seen that despite the 11 generated populations, there is a high degree of mixing between them, and the same 3 more consistent populations can be seen. The 3 distinct populations are Jacobin pigeon, Mondain and Salonta Giant.

## DISCUSSION

Evaluating genetic diversity indices in relation to breeding histories is a crucial aspect when assessing the genetic resources of breeds. This study aimed to elucidate the phylogenetics and genetic diversity of giant domestic pigeon breeds by analyzing a specific section of the mtDNA COI region. To achieve this, we included 276 individuals from 19 domestic pigeon breeds originating from the Carpathian Basin and Iraq.

### Phylogeny of Giant Domestic Pigeon Breeds, in Particular in the Carpathian Basin

Most giant domestic pigeon breeds can be traced back to the Mediterranean region, but the origins of the giant pigeon breeds from the Carpathian Basin remain unclear. One possible explanation is that they are descendants of the giant breeds historically known from the Mediterranean. The Carpathian Basin, located at the heart of Europe, is a geographically diverse area with numerous contact zones and is often considered a hotspot of biodiversity due to its high species and population diversity (Kajtoch et al., 2016; Mráz and Michał, 2016). Given the ongoing debate about the biological relevance of distinguishing between pigeon breeds and breed groups (Stringham et al., 2012), it is prudent to first compare the known breed groups included in the study before exploring potential regional relationships and geographical origins.

Over the past 3 decades, numerous studies have explored the origins of pigeon domestication. A popular hypothesis suggests that the rock pigeon was independently domesticated at nearly the same time in several locations within the Mediterranean basin and the Middle East (Johnston, 1992; Johnston and Janiga, 1995; Shapiro and Domyan, 2013). However, recent analyses indicate that the Levant region (Syria, Iraq, and Palestine) was the primary center of pigeon domestication (Hernández-Alonso et al., 2023). This region is closely linked to historical trade routes and empires. Similarly, the Carpathian Basin has played a significant role throughout history as a major agricultural source, destination, and transit area due to its strategic geographical location. Live animals were a crucial component of trade in this region (Nicodemus, 2014).

Haplotype diversity measures the genetic variation between two randomly selected alleles and their ratio. For domesticated animals, as with wild ones, a higher haplotype diversity value indicates greater genetic variation within the breed, reflecting a rich history of species development (Naderi et al., 2007; Dyomin et al., 2017; Yang et al., 2018). In the genetic diversity indices of pigeons categorized by origin (Table 4), the Mediterranean pigeon group (Carnao, Mirthys, Mondain, Runt) displayed the highest haplotype diversity (H_d_ = 0.576 ± 0.078), suggesting a long breeding history for giant pigeon breeds. This is particularly notable when compared to the American (H_d_ = 0.191 ± 0.093) and Hungarian Squab (H_d_ = 0.105 ± 0.092) breeds, which have fewer elements. This finding supports the hypothesis that the development of giant pigeon breeds originated in the Mediterranean region, where they were most extensively developed, as historical records indicate that pigeons were bred and consumed for meat in the Roman Empire (Varro, 1912). Although early pigeon breeding is well-documented in the American, Asian, and Mediterranean regions, there is no surviving evidence from the Carpathian Basin, leaving the origins of pigeon breeding there uncertain. Our results indicate that, despite variations in sample sizes, the Great Hungarian Plain pigeon group also exhibited high haplotype diversity (H_d_ = 0.486 ± 0.071), whereas the Asian group showed a lower value (H_d_ = 0.305 ± 0.057). This suggests that giant domestic pigeons may have been present in the Carpathian Basin for a considerable period and were not introduced only in the last one or two hundred years, as some written records suggest. Additionally, high haplotype diversity values observed in traditional breeds like Jacobin, Salonta Giant, and Hungarian Giant likely reflect the earlier development of these breeds compared to others. In this study, the number of polymorphisms was quite different between the traditional breed groups, which was probably due to the different numbers of elements between the groups, but in one case this value is not directly proportional to the change in the number of elements. For example, the Structure pigeons group includes 1 breed (Jacobin pigeon) in this study, and the number of elements is only n = 10. Nevertheless, we measured a remarkably high polymorphism diversity in this group (120). With a lower number of elements, we detected a similarly interesting result during the cluster analysis in Figure 5, 3 populations showed a definite genetic structure, which were the Jacobin pigeon, Mondain and Salonta Giant. Mondain and Jacobin are internationally widespread breeds with significant numbers of individuals. Salonta Giant is a localized breed with small numbers, so it is possible that the small population size may have helped to maintain a consistent genetic character.

This study found similar haplotype and nucleotide diversity values for the Utility and Squab pigeon groups, likely because Squab pigeon breeds were primarily developed from breeds within the Utility pigeon group. Due to this genetic relationship, Utility pigeons may continue to exhibit high diversity within domestic pigeons. Literature indicates that closely related squab breeds, despite having fewer samples, display comparable diversity values. For comparison, Firyal et al. (2014) examined Pakistani domestic pigeons and rock pigeons (*Columba livia*), noting an overall nucleotide diversity of 0.6%. Their analysis, conducted at the species level, considered the total nucleotide diversity of *Columba bollii* and *Columba junoniae* (0.16), leading to the classification of the Pakistani domestic pigeon as an independent species (Marrero et al., 2008; Firyal et al., 2014). Based on the results in the Supplementary File 4 and 5, the Breeds from the Great Hungarian Plain group have negative Fst values compared to both the Mediterranean and Hungarian Squab breeds, which is due to the fact that there is more variation within the groups than between the two groups, suggesting a small genetic distance, which refers to the relatedness of giant pigeons, regardless of their region of origin (Gerlach et al., 2010). Due to the strict regulations for the registration of purebred dogs, in which modern breeds are practically closed breeding populations, breeds are separated by large genetic distances (Parker et al., 2004; Vaysse et al., 2011). Domestic pigeons, on the other hand, do not have such pronounced genetic boundaries due to their mixed origin (average Fst between dog breeds Fst= 0.33, while this is the case for domestic pigeons: Fst = 0.24) (Stringham et al., 2012). Based on the genetic distance values between the breeds, the low genetic distances shown in Supplementary File 5, support the hypothesis that these breeds share a common ancestor. For the Blue Sovater breed, all values are statistically significant (p<0.05), likely because it is a relatively new breed that has not yet developed a distinct genetic profile. The Hungarian Domestic pigeon, which is not yet officially recognized, also shows a close relationship with other Hungarian breeds, such as the Buga pigeon, suggesting a shared ancestral lineage. Caution is warranted when interpreting the data for the Texan pigeon, as only one haplotype was detected, and the genetic diversity index values were low, likely due to the small sample size (n=9). The King and Carnao breeds show close genetic relationships with most other breeds, supporting existing literature on their common origins and interbreeding among these meat-type breeds (Eggleston, 1921). The AMOVA results obtained based on breed groups and the level of the breeds, indicate that these breeds exhibit distinct genetic profiles, justifying their classification as separate biological entities. Similar tests were carried out with Italian pigeon breeds, during which the individuals were examined in several different groups. In 2016 Bigi et al. were performed the comparison with breeds from other European countries, the condition being that the breeds included in the study had a common ancestor. Among the 427 individuals were roller and high-flyer pigeons, structure pigeons, frills, wattled pigeons, squab pigeons and poule-type pigeons. Based on their results, a smaller proportion of the observed total genetic variance, 21.34%, was formed between breeds, while 74.69% was measured at the level of individuals (Bigi et al., 2016). So, a small, but significant part of the genetic diversity was a characteristic that made the breeds unique and distinguishable from others even at the level of a group. The analysis was also performed by regrouping the breeds, but the AMOVA value did not change significantly. Compared to the Italian study, the obtained value in this study between breeds, was 30.52%, and between breed groups was 35.07% is significantly higher, which proves the marked genetic uniqueness of the breeds we studied. In our study, the intra-breed diversity surpasses inter-breed variation, the AMOVA results underscore the importance of maintaining traditional breed groupings and the biological distinctiveness of these breeds.

The values obtained by Principal coordinate analysis (PCoA) well support the distance values between the breeds compared based on the Fst value per pair, because on the PCoA Figure it was not possible to visualize either the Poule Type pigeon group or the Trumpeter pigeon group. Also, in the case of the comparison per breed in Supplementary File 6, the Blue Sovater (which also showed significant distance values in the Fst-based table) individuals mapped so far from the other breeds in the coordinate system that they could not be visualized. This also shows, as confirmed by the AMOVA analysis as well, that the breeds are most often clustered in one group, therefore differences are more likely to appear on the level of individuals. The comparison between by breeds, some of the Asian and Mediterranean individuals are farther apart from each other than the rest. The results based on the grouping according to the origin of the breeds, does not show a clear pattern, there is a high degree of mixing between individuals. The same structure cannot be said for Pacheco et al. (2020) on the Multidimensional Scaling diagram based on the pairwise Fst values, on which the different populations or breed groups are clearly separated, which are mostly in line with Stringham et al. (2012) and Shapiro et al. (2013) with the results obtained. According to them, this grouping pattern is a direct effect of continuous artificial selection. These differences are probably also due to the different breed group classifications, because in the literature mentioned above (Stringham et al.,2012; Shapiro et al.,2013; Pacheco et al., 2020) they all used the one used by the National Pigeon Association (**NPA**) of the United States of America, while in our study we used the breed group classification of the Entente Européenne Pigeon Breeds Association (**EE**). In this study all these results highlight the outstanding genetic diversity present in the domestic pigeon species, which is also supported by the phenotypic richness of the species.

In case of haplotype distribution, Figure 2 and in the network Figure (Figure 3A) the grouping by traditional breed groups clearly shows that the Squab pigeons are descended from the Utility pigeons. The Squab group peculiarity of this group of pigeons is that, unlike other groups, only one main criterion, meat production ability, is used as a criterion for classification. The Squab breeds (Hubbel and Mirthys) belong to the common haplotypes with the Utility pigeons, which is logical, since these breeds are younger compared to the others, and they are crossed from these types of Utility breeds. The Mediterranean region always has a common haplotype with the American region, which confirms that the majority of the American breeds developed from a Mediterranean base and that there is a relationship between them. This is well illustrated by the example of the Carnao pigeon, as it was originally bred in France for meat production (Levi, 1986; Sell, 2009), it was developed from the early Carrier archetype (Moore, 1735), which is probably the ancestor of the English Carrier, Scandaroon and Racing Homer pigeons. Later, around 1900, the breed was brought to the United States, where its phenotype was greatly changed as a result of crossbreeding, so that today's American version is much larger than the French version (Pacheco et al., 2020).

In 2012 SNP-based diversity studies by Stringham et al. showed similar results, with the traditional breed groups clearly outlined in the tree. In the network Figure 3, 219 individuals from all groupings were detected mixed in Hap_2. In 2019, Biray studied the genetic diversity of domestic pigeon breeds in Turkey, where the vast majority of individuals included in the study belonged to one haplotype, similar like our study and concluded that domestication of pigeons probably occurred in one center. Also, other haplotypes associated with singletons to larger haplotypes are usually the result of more recent mutational events (Biray, 2019).

In the Figure 3, the Jacobin in the Structure pigeon group is on a markedly separate evolutionary branch which can primarily be traced back to the history of its development (He and Jia, 2015) and unique phenotypic characteristics. In some previous literature, a relationship was established between the Jacobin pigeon and the Danish Tumbler pigeon, as well as the Old Dutch Capuchine, breeds that are quite different from each other (Moore, 1735; Levi 1986, 1996; Stringham et al., 2012). The Jacobin pigeon's unique phenotypic character, the oversized neck crest, which is not shared by any other domestic pigeon breed, made it difficult to improve the breed through crossbreeding, which encouraged strict inbreeding to maintain the genetic integrity of the Jacobin pigeon's distinctive neck crest. A good example of this is the case of a Canadian breeder who, in the interests of breed improvement, wanted to transplant the long neck of the English Carrier breed into the Jacobin. It took him 20 yr to produce birds with the standard appearance from this cross-bred lineage (oral communication I. Juráskó, Jacobin breeder).

In the Supplementary File 8 and in Figure 3B shows the haplotype distribution among the examined individuals according to the origin of the breeds. It can be seen that the breeds of the Carpathian Basin are very diverse despite the equalized sample numbers and have many local haplotypes. This geographical region is still of great importance in the development of biodiversity today, as Europe's most remarkable and largest european roller (Kiss et al., 2020), goosander mergus merganser (Kajtoch and Bobrek, 2014) brown bear, gray wolf and Eurasian lynx populations are also found here (Cristian-Remus Papp et al., 2022), and many continental areas played a significant role as a refuge area, in the formation of arctic and arctic-alpine taxa in the Pleistocene (Bálint et al., 2011). The number of phylogeographic/phylogenetic studies is increasing, revealing significant genetic and taxonomic diversity. According to the studies, the isolation of the populations in the major distribution area of the Carpathians played a major role in the high degree of plant and animal biodiversity developed here in the case of the Carpathian basin and the Carpathian Mountain range (Bálint et al., 2011). Our findings suggest that the high diversity values observed in Hungarian pigeon breeds may result from their early emergence in this geographical region. The ancestors of these breeds have likely been evolving in the Carpathian Basin for an extended period, potentially giving rise to an indigenous, independent population. Consequently, the Carpathian Basin—and Hungary within it—might have served as a bridge between Asia and the Mediterranean in the spread of domestic pigeons. This hypothesis is supported by the closer genetic relationship of Carpathian Basin haplotypes to those found in Asia, suggesting a possible Asian origin. If the Carpathian Basin breeds had originated from the Mediterranean region, we would expect a similar genetic pattern; however, our results indicate a greater genetic overlap with the Asian region.

### Assessment of the Iraqi Domestic Pigeon Breeds

In the case of the Iraqi breeds, based on the results, the genetic diversity is much lower than in the other breeds. The genetic distance with the Fst values between the Iraqi breeds (Supplementary File 4) is relatively low, and the genetic diversity within the breeds was also moderate (Supplementary File 3), the reason for which could be, for example, the very disciplined, closed breeding practice.

From the PCoA figure according to the breeds (Supplementary File 6), it is clear that the 2 Iraqi breeds examined are located next to each other, which also supports the uniformity of the breeds, and it can also be seen that the Bokhara Trumpeter breed, which is also of Asian origin, is located relatively close to the breeds.

If we compare the Iraqi results with the Asian breeds and groups, from the dendrogram (Figure 2), and the network figures (Figure 3) it is also clear that the formation of the Structure pigeon can be traced back quite a long time, as its unique haplotypes can be found on the branches closer to the trunk. Furthermore, the Asian haplotype group is located at the bottom of the tree, which clearly shows that the Asian breeds evolved on earlier branches, they are probably older breeds than the Mediterranean group, whose haplotypes are shown at the top of the Figure. Tumbler has some unique haplotypes in the Figure 3A, which include the Iraqi Red and Raabi pigeon. In terms of unique haplotypes, Iraqi Raabi has only 1 and Iraqi Red has 3 unique haplotypes. The Iraqi Red pigeon, which holds significant cultural importance in Iraqi society, is primarily bred in central and southern Iraq, particularly around Baghdad. There, it is known as the Baghdad Red pigeon (Shao et al., 2020). Nothing proves its extreme popularity better than the fact that the price category of Iraqi Red pigeon pairs can range from 10 to 50 dollars. Also, regarding the Iraqi Raabi breed, according to the breeders the Raabi Pigeon has cultural and historical significance in Iraq. The breed's association with the martyrdom of Imam Hussein has reinforced its cultural significance, especially among Shiite Muslims. Above all, it is important for breeders to know the genealogy of pigeons. The Raabi Pigeon is the result of selective breeding practices carried out over generations. The breeders’ pigeons with the desired characteristics such as color, size and plumage are carefully selected to produce offspring that meet the desired look. Each diversity indices clearly reflects the efforts of Iraqi breeders to maintain the homogeneity of their breeds. It also supports their reports from the sample collection, which indicate that preserving the pigeons' pure lineage is considered more crucial than their phenotypic traits. Although phenotypic appearance is important in the selection of breeding animals, only individuals of known and pure pedigree are accepted as breeding candidates. Phenotypic selection comes afterwards. The high social esteem of the Iraqi Red pigeon, along with our collected reports on Iraqi breeds, suggests that despite its widespread popularity, breeders are dedicated to preserving purebred flocks of this variety, as reflected in our results. This commitment to uniformity is evident in the perspectives of Iraqi breeders, who view raising Iraqi Red pigeons as a treasured tradition passed down through generations. Many farmers are deeply invested in this hobby, dedicating significant time and effort to selecting high-quality birds and maintaining the breed. The passion for Iraqi Red pigeons is deeply embedded in Iraqi culture, with a strong focus on preserving the breed's unique qualities and characteristics.

### Effect of Different Domestic Pigeon Breeding Practices in Central Europe and the Middle East on the Genetic Diversity

As far as pigeon breeding practices are concerned, in North America and Europe breeds are bred according to written standards and strive for uniform phenotypic appearance. In Iraq, on the other hand, there are no written standards, but breeders take the knowledge of pedigrees very seriously.

High diversity can be influenced by many factors. According to Pacheco et al. (2020), the breeds created as a result of the recent hybridization of different breeds can be expected to contain several breed groups, since a hybrid breed is on a branching trunk, its location tends to change a lot, which may be true for the younger Squab pigeon types. Our previous study confirms the mixing between breeds, because in our study we examined racing pigeons and squab pigeons using chicken DNA chip genotyping, where it was clear that there was a large genetic overlap between the utilization types, because these utilization branches were not yet significantly separated (Balog et al., 2023). In this study, compared to all other breeds, the number of polymorphisms and genetic diversity indices of the Iraqi breeds were low, and the two breeds had fewer unique haplotypes than the other breeds. However, based on the geographical proximity to the domestication area, high diversity values would be expected. One possible explanation for this result is that is that those Iraqi breeders who prefer purity of origin over uniform appearance achieve lower genetic diversity in their pigeons. In contrast, in breeding practices that emphasise phenotypic traits, crossbreeding can often occur because when breeders want to improve a trait, the quickest solution may be to mate their own pigeons with pigeons that have already developed the desired trait. Thus, breeding practices that focus on uniformity of appearance take less account of purity of pedigree and can lead to greater genetic diversity in the breed or population.

Shapiro et al. (2013) argue that differences between breeds are often attributed to macroevolutionary changes rather than intra-species variations. Their study suggests that the evolution of pigeon breeds, similar to many domesticated species, is unlikely to follow a purely linear or hierarchical path (Stringham et al., 2012; Shapiro et al., 2013), indicating a need for a new, non-classical classification system to address the taxonomy of domestic pigeons. Additionally, breeders frequently exchange and cross pigeons, which can lead to the transfer of mitohaplotypes between unrelated breed groups, thereby reducing genetic differences (Biray, 2019). Our results support these findings for North American and European breeds and also reveal the influence of another pigeon breeding tradition in Iraq.

## CONCLUSIONS

Our study offers the first comprehensive insight into the genetic diversity and phylogenetics of giant domestic pigeon breeds used or potentially used in the squab industry. It reveals high genetic diversity and significant interbreeding among these breeds, suggesting a complex history of genetic mixing. Although traditional breed classifications have a genetic basis, their distinct genetic identities have not fully emerged. Our findings support traditional breed groupings, with the Carpathian Basin exhibiting high genetic diversity comparable to the Mediterranean and Asian groups. Results from the present study validate the use of the mtDNA COI marker but suggest future studies should explore other markers, as there are still many pigeon groups and populations worth investigating. The study confirms the significance of breed-based segregation and the value of pedigree systems in maintaining breed uniformity, particularly among Iraqi breeds.

## ACKNOWLEDGMENTS

The authors would like to thank Nóra Ninausz for her help in laboratory work. The authors are grateful to Bíborka Sipos for her help in collecting the samples. The authors express their gratitude to the local pigeon breeders in Hungary, Romania and Iraq for their invaluable contribution to providing the samples.

This work was supported by the ÚNKP-22-3-1 New National Excellence Program of the Ministry for Culture and Innovation from the source of the National Research, Development and Innovation Fund. The study was supported by the University of Debrecen Program for Scientific Publication.

Katalin Balog was supported by the PhD Excellence Scholarship from the Count István Tisza Foundation for the University of Debrecen.

Thanks, are also due to Erzsébet Fejes for language proof-reading and anonymous reviewers for their valuable comments on an earlier draft of the manuscript.

Author Contributions: Zoltán Bagi: Conceptualization, Sampling, Methodology, Review and Editing; Katalin Balog: Sampling, Writing - original draft, Laboratory work, Analysis, Data Visualization; Ali Al-Sallami Wadday: Sampling, Review and Editing; Baraa Akeel Al Hasan: Review and Editing; George Wanjala: Analysis, Data Visualization, Review and Editing; Szilvia Kusza: Conceptualization, Review and Editing; Péter Fehér: Laboratory work, Review and Editing; Viktor Stéger: Review and Editing.

## DISCLOSURES

The authors declare the following financial interests/personal relationships which may be considered as potential competing interests: Katalin Balog reports financial support was provided by Ministry for Culture and Innovation from the source of the National Research, Development and Innovation Fund (Hungary). If there are other authors, they declare that they have no known competing financial interests or personal relationships that could have appeared to influence the work reported in this paper.

## Appendix Supplementary materials

Image, application 1

Image, application 2

Image, application 3

Image, application 4

Image, application 5

Image, application 6

Image, application 7

Image, application 8

## Footnotes

Supplementary material associated with this article can be found in the online version at doi:10.1016/j.psj.2024.104310.
