# Supplementary material for: MtDNA genetic diversity and phylogeographic insights into giant domestic pigeon (Columba livia domestica) breeds: connections between Central Europe and the Middle East
Source: Poult Sci. 2024 Sep 7;103(12):104310. doi: 10.1016/j.psj.2024.104310 (PMC11458985; doi:10.1016/j.psj.2024.104310)
Supplement: Supplementary file 1 [file mmc1.pdf]

PIGEON DOMESTICATION: A PHYLOGEOGRAPHIC STUDY

MtDNA Genetic Diversity and Phylogeographic Insights into Giant Domestic Pigeon (*Columba livia domestica*) Breeds: Connections Between Central Europe and the Middle East

K. Balog, A. S. Wadday, B. A. Al-Hasan, G. Wanjala, Sz. Kusza, P. Fehér, V. Stéger, Z. Bagi[1]

Supplementary File 1. Description of the domestic pigeon breeds examined in the study

Archaeological evidence suggests that the rock pigeon (*Columba livia*) was domesticated for the first time in the Neolithic period in the Middle East, where it was initially used as a divine symbol and also as a messenger during wars (Driscoll et al., 2009; Vickrey et al., 2015). The domestication process of the pigeon probably started in the Middle East in the years 6000–4000 B.C. (Szűcs and Szécsényi, 1965, Driscoll et al., 2009; Vickrey et al., 2015).

Later, in the 1800s, Charles Darwin conducted successful breeding experiments on various pigeon breeds to prove his theory of natural selection and the role of artificial selection (Darwin, 1859). As with other domesticated animals, the high morphological diversity of domestic pigeons is due to the crossing of historical kingdoms and trade routes (Pacheco et al., 2020). However, according to Casanova (2013) and Pacheco et al. (2020), pigeon breeders have recorded little information on which breeds were crossed or selected to create new breeds, therefore the information on pigeon breeds is much less documented than that regarding e.g. cattle or horses. In this supplementary we have collected the available written literature on the 19 breeds examined in this article.

1. **Bokhara Trumpeter**

Category:
Traditional breed group: Trumpeter pigeon
The origin of the breed: Asian pigeons

In ancient times the Uzbek city of Bukhara was a well-known trading city and the gateway for many products from Asia. In the numerous mosques, many pigeons were traditionally kept. The pigeons were often in the possession of merchants accompanying the itinerant caravans that at that time plied between various countries. They bought or traded pigeons in exchange for other merchandise. One of the earliest individual imports was brought to Britain by the well-known Irish author George Ure. In his book “Fancy” he describes the arrival in Ireland of some Bokhara Trumpeters from Moscow in around 1850 A.D. A few years later another import from Russia took place by sea. John Moore in his famous Columbarium, published in 1735 – the world’s first real Fancy Pigeon book – gave a description of the “English Trumpeters”, so called because the Bokhara Trumpeter was still completely unknown at the time. When the trade war with the Emir of Bukhara ended in 1873, Russian pigeon merchants could finally do business without hindrance. A number of these pigeons ended up with interested German pigeon breeders, and also in the Paris Zoo. These individuals' descendants came into the hands of the well-known Jacobin breeder and pigeon judge, Herbert Smith. In the meantime, there was also

[1] Correspondence should be addressed to Zoltán Bagi, Centre for Agricultural Genomics and Biotechnology, University of Debrecen, 4032, Debrecen, Hungary, Tel:
+36 52 508 444 / 88521, 68304, Email: bagiz@agr.unideb.hu

great American interest in these pigeons: the American brothers Al and Brian Grace, both well-known Jacobin breeders, were very interested in Bokharas, but really top animals were out of their reach, thanks to the high prices. This gave them the idea to cross their Jacobins with the Bokharas already in their possession. The long feathers of the Jacobins perfectly matched the plumage of the Bokharas, who were also provided with long feathering, and besides triggering energetic renewal and revitalisation, the cross also served the advancement of the breed and gave rise to a number of new colours. These pigeons are named for the sound of their voice - the Dutch and the German fanciers call it "drumming", the English and American fanciers "trumpeting". The Bokhara Trumpeter is a large, squat bird; it is well-developed both in length and width, and belongs to the larger pigeon breeds; however, they are not Runts, as some mistakenly think. The loose feathering makes them appear more robust than they really are. The very deep physique is striking; the breast is very broad, well rounded and is of great importance in obtaining the right depth. In really good specimens, a sitting bird must give the impression that it is laying, because of the rich and abundant plumage of the legs, hocks and feet. Ring size is 12 mm.

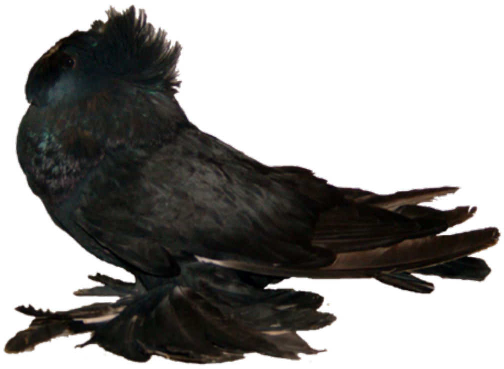

Figure 1 Bokhara Trumpeter
Source: https://www.roysfarm.com/bokhara-trumpeter-pigeon/

2. **Blue Sovater**

Category:
Traditional breed group: Utility pigeons
The origin of the breed: Breeds from the Great Hungarian Plain

This Hungarian breed was developed in the Great Hungarian Plain, more precisely in the town of Hajdúszovát. It was bred by crossing Carrier pigeon, Show Homer and King breeds and recording the external characteristics of the offspring in the years 1975-1986. Recognized as an independent breed, it was included in the list of Hungarian breeds in 1987. The main characteristics of the appearance: larger than medium size, well-muscled, almost horizontal posture, only blue, barless color recognized; meat type utility breed. The average body weight of the adult males is 700-900 g, and that of the hens, 600-700 g. Ring size is 10 mm.

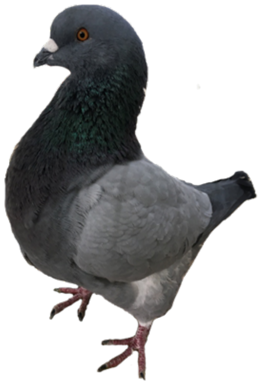

Figure 2 Blue Sovater

3. **Buga pigeon**

Category:
Traditional breed group: Utility pigeons
The origin of the breed: Breeds from the Great Hungarian Plain

It is a native Hungarian breed, especially common in the Southern Great Plain region. The breed is larger than average, with a stocky build. A powerful, foot-feathered, creasted bird, with elevated posture and a body length of 35–40 cm. The breed has a heart-shaped pattern, in yellow, red, blue and black. According to some, the breed's ancestors were bred from large, smooth-headed, strong-boned, well-bred, disease-resistant „Turkish Pigeons" – which is a theoretical, unproven breed – brought by the invaders in the period following the Turkish occupation. It is more likely to assume that it developed as an independent breed from the descendants of the Hungarian Giant Pigeon and the highflyer pigeons of Turkish origin bred in the Szeged area. Nowadays, it is considered by many to be one of the most beautiful Hungarian pigeon breeds. This large, colorful, large foot-feathered pigeon attracts other pigeons in the area with its snapping flight. One of the main goals of the breed was to develop a large, disease-resistant and prolific pigeon that would provide the family with continuous fresh meat in addition to the animals raised in the backyard. Ring size: 12 mm.

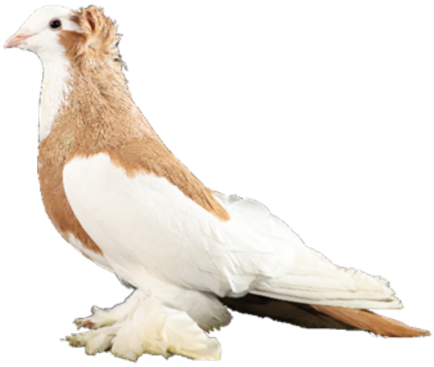

Figure 3 Buga pigeon

4. **Carnao pigeon**

Category:
Traditional breed groups Utility pigeons
The origin of the breed: Mediterranean group

Popular squab pigeon of Northern France and Southern Belgium. It was brought to Belgium from France, and later spread extensively in the United States. Originally it was a typical „field pigeon”, and it is thanks to this that its economic properties are still excellent today. According to earlier records, it was created by crossing French Mondain with the local feral pigeons, which were then subjected to consistent selection. During the selection, the reddest individuals were always preferred. Various breeds were also imported to the United States from Syria in 1954. These also included red and yellow Lebanese breeds, which are very similar to Carnao. Even the eyes and the nose bump are the same. These facts suggest that the origin of the French Carnao can be traced back here. The rationale can even be confirmed by the fact that France and Syria had lively trade relations with each other in the 16th, 17th and 18th centuries. It is an excellent profit breed, with excellent quality skin and brilliant meat forms. It is a very prolific, first-class breed, with a calm temperament. Over time, it also became popular as a fancy pigeon. The Carnao is a low-standing, stocky, well-muscled type of meat type utility pigeon. Ring size 10 mm.

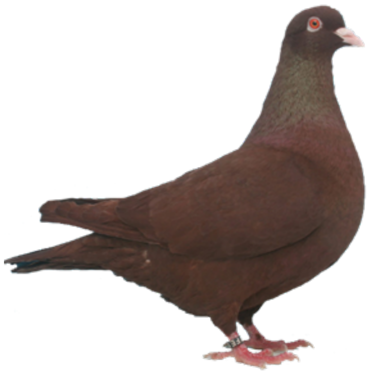

Figure 4 Carnao pigeon
Source: https://backyardpoultry.iamcountryside.com/poultry-101/pigeon-types-rollers-to-racers/

5. **Hubbell**

Category:
Traditional breed group: Squab pigeons
The origin of the breed: American group

The Hubbell breed was bred in the 1910s in California, USA, from the King utility breed, using the Texan and Mondain breeds. The Hubbell was introduced to Europe in 1983 and spread very quickly to industrial squab pigeon farms. Initially bred mainly in white, there are now coloured (recessive red, black, silver) and even autosex flocks. Genetically, the white color variant is considered the most uniform, with the white feather color having an advantage in terms of marketability due to the associated light skin color. The general characteristics of the breed are a calm character, a sufficiently (but not too) large body, an intense developmental vigor, vigorous rearing, excellent reproductive and rearing ability, a full body and excellent fleshiness, especially the exceptional development of the pectoral muscles, which is why the breed is often referred to as double-breasted. Compared to other squab pigeon breeds, the proportion of breast meat is around 10–15% higher. The live weight of adults is between 650 and 800 g, which means that they are not among the heaviest meat pigeons, which is an advantage due to their lower life-sustaining energy needs. It rears 15-17 chicks per breeding pair per year. Their slaughter yield is around 70-72%. The ring size is 10 mm.

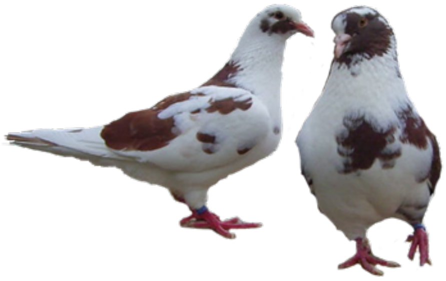

Figure 5 Hubbel
Source: https://www.pigeontype.info/2020/12/25/hubble-farming-pigeons/

6. **Hungarian Chicken pigeon**

Traditional breed group: Poule type pigeons
The origin of the breed: Hungarian squab group

The Hungarian Chicken pigeon was first officially described in 1970 by dr. István Péterfi, but based on the narratives, it has existed on the Hungarian Great Plain for much longer. It is an economically valuable breed, as it is an excellent fancy pigeon, and it is also suitable as a „nanny pigeon" for raising the offspring of breeds that are difficult to reproduce. They have no special needs, they are very viable, well-adapted and disease-resistant birds. It is characteristic of the breed that, regardless of their feather color, all pigeons have yellow irises, except the white ones. It is also characterized by its deep, relatively short, relatively strong trunk, which is held horizontally. The chest is slightly convex, proportionally wide, muscular, the shoulder blades are not visible, they are covered by the plumage of the chest. The adult body weight is 600–800 g. The Bánát and Vukovar Chicken pigeons come from the same stock as the Hungarian breed, which could later become separate breeds due to the modified national borders (Bánság-Bánát, like Vukovar, was part of historical Hungary), although significant differences have not emerged between them to this day. The description of the Banatian Chicken pigeon was taken from the Hungarian Chicken pigeon with the only difference that the breeding country and the color of the white's irises and eyelids were modified, and there is a minimal difference in the weight. The Vukovar Chicken pigeon, on the other hand, has already changed more, it is more similar to the King. Over the decades, of course, this change was caused by conscious cross-breeding. The Bánát and Vukovar Chicken pigeons are on the EE breed list, which means that they are also internationally registered. However, the Hungarian Chicken pigeon has not made it this far: it is only on the breed list of the Hungarian National Pigeon Breeding Association, although looking at its history, this breed could also be placed on the EE list. The ring size is 10 mm.

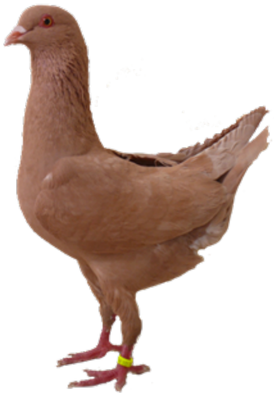

Figure 6 Hungarian Chicken pigeon

7. **Hungarian Cropper**

Traditional breed group: Cropper pigeons
The origin of the breed: Breeds from the Great Hungarian Plain

The origin of the breed is not clear. Some believe that its ancestors were brought from the East to the Carpathian Basin in the 16th and 17th centuries, but there is no evidence for this. The earliest depictions of pouter/cropper pigeons date from the 17th century in Western Europe, therefore it is more likely that pigeons from there have been shaped over the centuries by local tastes. Today it is considered one of the oldest and most characteristic Hungarian pigeon breeds. Even at the beginning, their bodies were already longer than that of an average pigeon, and their fanciers at that time were struck by their tendency to blow, their "piping" and small puffs. This gave the pigeons an interesting look, so their owners started blowing their pets, and selected only those that produced larger and larger crops (also known as the ingluvies) due to the artificial inflation, therefore they were prone to blow their own crop. At that time, even though the breed was very diverse, they were not completely uniform in shape and form. In the 1800s, when their breeders got to the point where standardization took place, the breed was changed to birds with smooth heads and smooth legs, and they prioritized specimens with shorter legs and a deeper posture, with as long a body as possible and a rounded-strong inflection. At that time, these pigeons were already called „Hungarian Pouter". Conscious color breeding only began in the early 1900s. Around 1910, the Hungarian Cropper began to be exported to Germany for the purpose of backcrossing, in order to save the Old German Pouter breed. It worked out so well that later there were disagreements about whether or not Hungarian Cropper and Old German Pouter are the same breed. Fortunately, thanks to the exact background of the breed in the literature, clear evidence indicated that there are differences between the two breeds. Currently available in 8 colors, the Hungarian Cropper has also been added to the breed list of the EE. The breed is very large, mainly long (minimum 60 cm). This domestic pigeon breed has the largest wingspan in the world. The largest measured wingspan is 124 cm. Other unique features are the curved, sabre-shaped form of the primary feathers and the short, wide-footed, huge, round balloon-blowing type of pouter. The latter is unique even in a state of rest with primary feathers lying on and extending beyond the tail feathers. The ends of the primary feathers cross slightly beyond the tail feathers. Its body weight at an adult age is at least 850 g. The ring size is 9 mm.

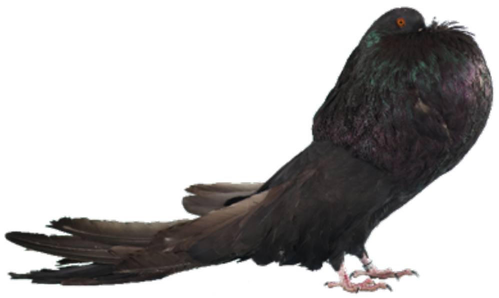

Figure 7 Hungarian Cropper

8. **Hungarian Domestic pigeon**

Traditional breed groups: Utility pigeons
The distribution of the breed: Carpathian Basin breeds
The origin of the breed: Breeds from the Great Hungarian Plain

It is not an officially recognized, standardized breed. It is widespread in Transylvania (currently Romania, but formerly part of the Kingdom of Hungary), where it is mostly kept as a „nanny pigeon" (raising chicks of other breeds) not bred for pigeon shows. Phenotypically it is a very variable group. Their body size is larger than the average pigeon body. Most specimens are white, but there are also variegated and colored ones. They can be with or without a comb; however, they are always characterized by large foot feathers. The name Hungarian Domestic pigeon was also applied to the Hungarian Giant pigeon in the early 1900s. It is also very similar to the Hungarian Peasant breed in many ways. Transylvanian breeders, however, know these breeds and clearly distinguish the Hungarian Domestic pigeon from them. According to our hypothesis, they may be survivors of the early type of the "Turkish Pigeon" breed circle, which has been preserved in isolation in the eastern corner of historical Hungary for the past 100 years. This breed is not officially registered in Hungary. The ring size is not known.

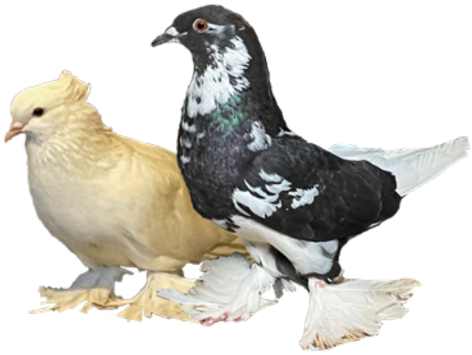

Figure 8 Hungarian Domestic pigeon

9. **Hungarian Giant pigeon**

Traditional breed group: Utility pigeons
The origin of the breed: Breeds from the Great Hungarian Plain

One of the most characteristic and best-known Hungarian pigeon breeds. Also known as the queen of the pigeons. There are no certain data on the origin of the breed, but even in the 1930s it was referred to as the „Turkish Pigeon". This was thought by many breeders to refer to the origin of the breed, but recent research has uncovered literature that describes the breed's ethnographic collection, which has given it this folk name because the breed was called a

Turkish pigeon because its appearance resembled a Turkish pasha, with its large "slippers" (muffs) and "turban" (crest). The term "pasha" refers to a high-ranking official in the Ottoman military and civil administration, including provincial governors. Therefore, the pigeon's name likely derived from its resemblance to this traditional and distinctive attire. The contemporary literature mentions that a type which is known as the „Turkish Pigeon" is a pigeon with a bumpy nose, which, based on literature, cannot be related to the Hungarian Giant by origin or kinship. Our extensive research in the Balkans, Turkey, Iraq, and Iran, including consultations with pigeon breeders and academic researchers, has shown that Turkish pigeon breeding culture does not favor large, heavy pigeons; instead, Turkish breeders prefer birds suited for flight. Based on this evidence and existing literature, it can be conclusively stated that the Hungarian giant pigeon has no counterpart in Asia, nor has it ever had one. In the 1930s, this breed still had many versions (with and without foot feathers, peak-crested, shell-crested etc.), and then in 1960, the first unified breed description was completed. This breed, as its name suggests, has a giant body. Furthermore, it has a large shell crest and foot feathers. Its body length from the tip of the beak to the end of the tail is 45–50 cm. It has strong bones and a well-muscled torso. The body weight is 800–900 g. The ring size is 15 mm.

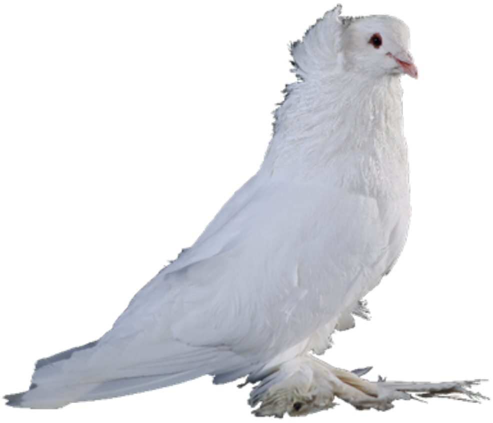

Figure 9 Hungarian Giant pigeon

10. Hungarian Peasant pigeon
Traditional breed group: Utility pigeons
The origin of the breed: Breeds from the Great Hungarian Plain

The Hungarian Peasant pigeon is a breed of domestic pigeon bred in Hungary. The breed was officially recognized in 2007; however, it has been known in the farm world in the southern cities of the Great Plain (Csongrád, Szeged, Szentes) for many decades. Not much is known about its origin. It has been a much liked breed because of its good breeding ability, tasty meat and full-bodied appearance. It is a large, strong, smooth-headed, foot-feathered pigeon with a not too long body and an almost horizontal posture, and it exists only in a snow-white version. Its body weight is 450–600 g. Ring size: 12 mm.

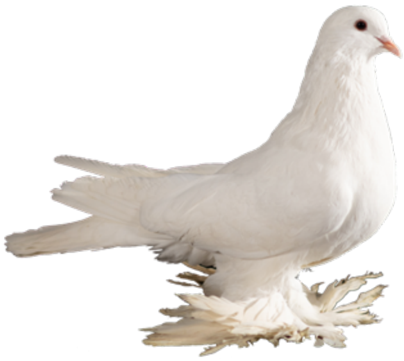

Figure 10 Hungarian Peasant pigeon

11. Iraqi Raabi pigeon
Traditional breed group: Utility pigeons
The origin of the breed: Asian breeds

The Raabi pigeon is a breed that is primarily raised in the southern provinces of Iraq, particularly in Najaf. It is closely associated with the martyrdom of Imam Hussein, a revered figure in Shia Islam and the grandson of Prophet Muhammad. According to religious traditions, the original color of this pigeon was white, but it turned red after the martyrdom of Imam Hussein. The Raabi pigeon is relatively heavy, weighing around 750 g on average, which makes it unable to fly. The presence of feathers around the legs further inhibits its ability to fly, and most breeders prefer lighter-weight pigeons as they are not typically consumed. It has been observed that most breeders prefer the red color of the Raabi pigeon, influenced by religious reasons. However, when left to mate naturally, the offspring tend to revert to white color. Some individuals have also been observed with white and yellow plumage. The Raabi pigeon is recognizable by its distinct features, including its long white beak and the presence of a crest of feathers covering the head. The body of the bird is relatively long compared to its overall size. Additionally, all Raabi pigeons have feathers covering their feet, giving them a medium-length appearance. In terms of behavior, the Raabi pigeon is characterized by its vocal cooing, which resembles laughter or trumpeting. The ring size is not known.

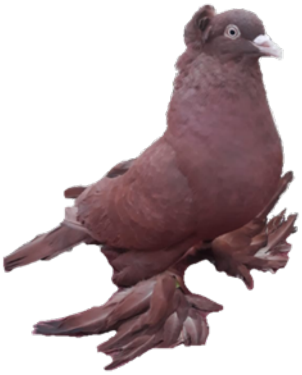

Figure 11 Iraqi Raabi pigeon

12. Iraqi Red pigeon
Traditional breed group: Tumbler pigeon
The origin of the breed: Asian breeds

The Iraqi Red pigeon, also known as the Baghdadi Red pigeon, is a breed of bird that holds an especially important cultural significance in Iraqi society. The body of the Iraqi Red pigeon

exhibits two colors. The most common color is red, followed by golden yellow, black, and blue. The presence of white color is consistent across all variations, but its distribution is more prominent in the head area, constituting approximately 6% of the bird's body. In terms of physical characteristics, the average body weight of the Iraqi Red pigeon ranges from 210 to 370 g. The beak length averages around 12.5 cm, whereas the wing length measures 20 cm. The head length is about 5 cm, and the tarsus length is around 3 cm. These measurements contribute to the breed's flying ability, with an average flying time ranging from 23 to 36 minutes. Distinguishing features of the Iraqi Red pigeon include a long white beak, which is a characteristic trait of the original breed. Additionally, the birds often possess a crest of feathers that covers the head, and their bodies are relatively long compared to their overall size. They are also characterized by having feathered feet of medium length. The breeding of Iraqi red pigeons is a cherished tradition passed down through generations. Many farmers have dedicated themselves to this hobby, investing time and effort in selecting and breeding high-quality birds. The passion for Iraqi Red pigeons is deeply ingrained in Iraqi culture, with a particular emphasis on preserving the unique traits and characteristics of the breed. The Iraqi Red pigeon is known by various names, these names often reflect the bird's physical appearance, color variations, or specific lineage. Farmers take pride in the different strains and lineages they maintain and continue to refine their breeding programs to enhance specific traits and colors.

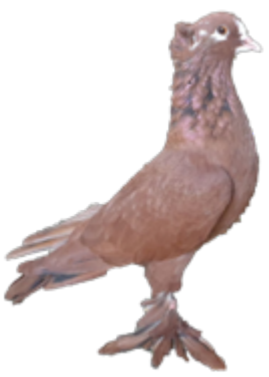

Figure 12 Iraqi Red pigeon

13. Jacobin
Traditional breed group: Structure pigeon
The origin of the breed: Asian breeds

Jacobin pigeons originated in Asia, and the breed went through many stages before the look of modern Jacobins was achieved. Breeders used selective breeding to develop Jacobin pigeons, a technique first employed as early as 1500. Then, explorers and traders brought Jacobins to Europe during the 16th century. Dutch sailors brought it from India to Europe around 1550, where the breed quickly spread in Western and Central Europe. It is also a very ancient breed in its native country. It was bred in England, but its present form was developed in the USA. The breed's name comes from the feather arrangements on their heads (known as a muff or cowl) that look similar to the hoods that Jacobin monks wore. This muff is quite particular, as the feathers grow backward and give the pigeon a ruffled look. The appearance of Jacobin pigeons is somewhat deceiving. While the fluffy feathers make the birds look big, they are actually small to medium, medium-sized pigeons with a steep stance and challenging posture. It is still a worldwide popular and widespread fancy pigeon breed. Breeders have developed Jacobins in multiple colors, including white, black, yellow, silver, red, and blue. This breed is quite complicated, because for example the females are selfish, and males can be aggressive, because they're highly territorial birds. During the breeding phase, the male will

claim the cage floor and take control of the area. As a result, others may struggle to eat because they may not have access to the food. Ring size is 8 mm.

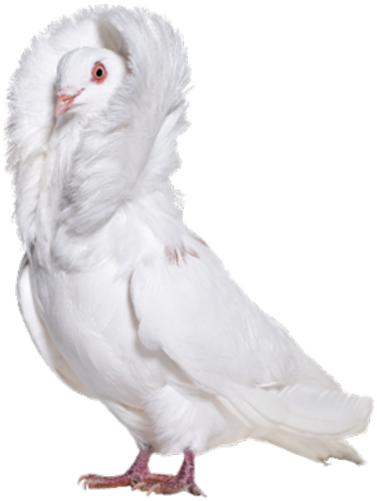

Figure 13 Jacobin pigeon
Source: https://www.envato.com

14. King
Traditional breed group: Poule type pigeon
The origin of the breed: American breeds

The King pigeon is the national breed of the USA, which is raised primarily for utility purposes. It was developed over many years of selective breeding. The breed was actually developed during the 1890s by crossing four pigeon breeds, namely Duchess, Homer, Maltese and Runt. The Duchess were selected for grace, Homer for alertness, Runt for body and size, and Maltese for compactness and style. The breeding of the King pigeon is attributed to the Vineland breeder Harry Baker. The first breed standard was formulated in 1915, which was accepted by the American Pigeon Breeders' Association. In the same year, the American White King Breeders Association was founded. So far only the white King has been mentioned, because colored pigeons were only included in the standard of the breed later, the silver ones in 1921, because they multiplied in large numbers. The first imported Kings arrived to Hungary in 1958. This date is also the beginning of the European spread. After six years, 258 birds were on display at the 1964 international pigeon show, whereas in 1969 there were already 795 of them. The breed has a wide, round, short body, which can be fit into an imaginary circle. All its body lines are curved, with a half-height stance, with straight legs placed in the center of the body; its round head continues in a strong thick neck, and its posture is therefore proud, graceful and elegant. The body weight of the breed varies between 800–960 g. The ring size is 10 mm.

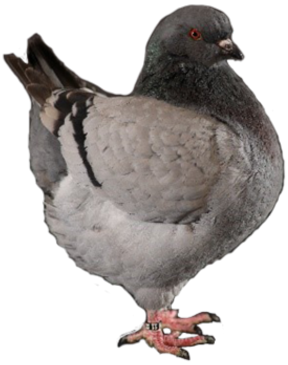

Figure 14 King

15. Mirthys
Traditional breed group: Squab pigeon
The origin of the breed: Mediterranean breeds

The livestock breeding company Grimaud Fréres and the INRA (National Agricultural Research Institute, France) jointly produced lines for the European market with the Europigeon breeding system. For the production of the first lines, the varieties white Carnao, white and silver King, Texan and Hubbell were used. In the selection of the maternal lines, the hatching rate and the egg-laying interval were ranked first; in the case of the paternal lines, body weight, growth vigor and constitution were the most important aspects. From these, the sustainable hybrids Le Mimas, Le Mirthys Blanc, Le Mirthys Coloré, Le Mirthys Pie and Le Titan Super Lourd were created. These hybrids raise 13-16 chicks per pair per year, which reach a slaughter weight of 650–750 g at the age of 28 days. In Europe, Mirthys is one of the most widespread squab pigeon hybrids in the meat production sector nowadays. Ring size is 10 mm.

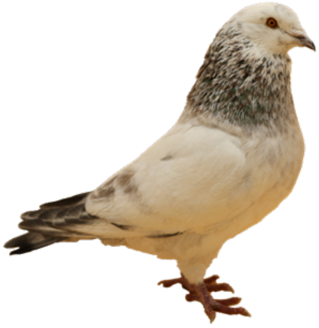

Figure 15 Mirthys

16. Mondain
Traditional breed group: Utility pigeon
The origin of the breed: Mediterranean breeds

Mondain is an old French breed that can be traced back to Montauban, Runt and Bagdad ancestors. Later, other breeds were also used for the final design of today's form. It was bred from the beginning of the 20th century and became an officially recognized breed in 1931. Two types are known: the American and the European French Mondain are actually different varieties with the same name. In terms of appearance, it has a very distinctive shape, very large, very heavy, short-legged; its chest is broad and protruding. Its posture when walking is almost horizontal. The expected weight is about 1000 g for hens and about 1100 g for males. Its shape

is short and its body is well-muscled. Its body length from the tip of the beak to the end of the tail is nearly 43 cm for males and 40 cm for hens. The ring size is 11 mm.

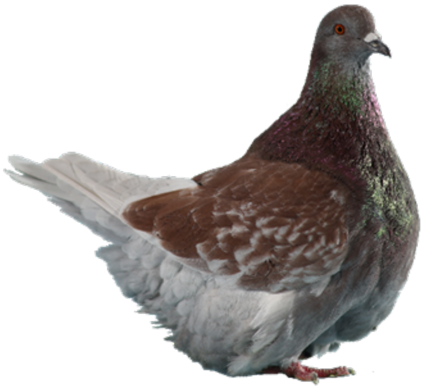

Figure 16 Mondain

17. Salonta Giant pigeon
Traditional breed group: Utility pigeon
The origin of the breed: Breeds from the Great Hungarian Plain

The Salonta Giant pigeon was formed in the eastern part of the Great Hungarian Plain (in the Partium area marked by the towns Nagyszalonta (Salonta), Arad and Nagyvárad (Oradea), currently Romania) in the last century. This originally Hungarian-bred pigeon breed has by now drifted almost completely to the brink of extinction. Contrary to false reports, the breed can still be found in a few places in the Partium area and nowadays also in Hungary. In the Hungarian specialized literature, information about the breed can be found in the older books, whereas the newer editions of pigeon breeding books hardly ever mention the breed. However, Romania registered it as an official Romanian breed in the last decade at the L'Entente Européenne d'Aviculture et de Cuniculture (EE). The Salonta Giant pigeon is a breed with a large body, strong structure and bones, double creasted and with a long and rich plumage. The body weight of males is 700–900 g, and that of the hens is 600–750 g. The chest is broad, convex, long and slightly forward. The chest circumference of males is 35–36 cm, that of hens is 33–34 cm. The back is long, straight, sloping backwards, the belly is wide, not very developed, with abundant plumage. It is a pigeon breed with good reproduction and breeding abilities, suitable for meat production and mass breeding. The ring size is 10 mm.

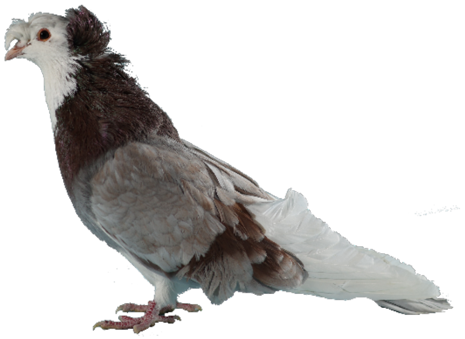

Figure 17 Salonta Giant pigeon

18. Runt pigeon
Traditional breed groups Utility pigeon
The origin of the breed: Mediterranean breeds

The Runt pigeon is one of the world's oldest pigeon breeds; its likely ancestors were large pigeons that were used as squab pigeons in the region of Campania, south of Rome. It is also called Romain Giant for its impressive size and weight. Runt pigeons appeared in the territory of Italy at around 2000 BC. The conditions of its selection and the breeds used are not exactly known. The Runt pigeon, although of Italian origin, has become a full-fledged French breed of pigeon, which is immediately recognizable by its large-sized, red-circled eye. It reaches a length of 55 cm. Adult males weigh up to 1300 g. The Runt is a large bird with a long massive beak and large feet. The Runt pigeon has long been an excellent breeding material and has been used to create new breeds. It is considered one of the ancestors of many utility pigeon breeds and squab pigeon hybrids. Ring size is 11 mm.

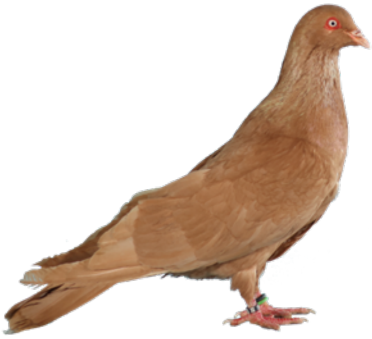

Figure 18 Runt pigeon

19. Texan
Traditional breed group: Utility pigeon
The origin of the breed: American breeds

Whereas the origin of most breeds of pigeons may be rather obscure, the origin of the Auto-Sex Texan Pioneer is very definite. This development project began in Houston, Texas in 1953 by Mr. Delwin V. James. The Texan Pioneer was developed to obtain the most efficient squab producer possible. Breeding was started from parents who are fast producers, good feeders, not overly large, and who have high disease resistance and a long producing period of life. The forerunner of the Texan Pioneer was called the Auto-Sex Texan. This was a loft name given them for record purposes only. The Texan was produced with an auto-sex factor through the use of Auto-Sex Kings and the French Mondains, giving a hybrid of 3/8 Auto-Sex King and 5/8 French Mondain. The perfect squabbing type was quickly recognized for its show quality. The Auto-Sex Texan Pioneer has tight feathering, broad breast, short tail and wings, and has a fairly straight back and tail line. The mature birds weigh between 800–950 g in prime condition. The Auto-Sexing factor allows the identification of cocks and hens at any time from hatching date on. The hens are long down and will feather out faded colors of blue, black, ash-red, or "T-check" pattern ash-red, whereas the cocks are short down, white with some flecking of colored feathers on the neck and some about the body. In the past few years, the Recessive Red color

has emerged in the Texan Pioneer Breed. The Auto-Sex Texan Pioneer breed was recognized by the National Pigeon Association in 1961 as a new/pure breed. The first specimens were brought to Europe and Hungary in the early 1970s, and within a few years the breed became extremely well-known throughout the world, and many breeders breed them as fancy pigeons today. The ring size is 10 mm.

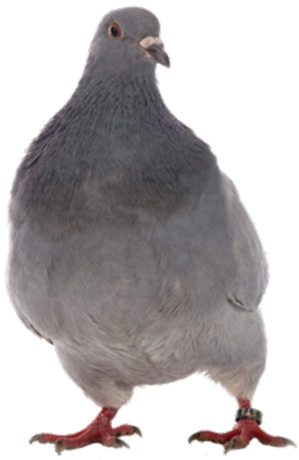

Figure 19 Texan
Source: https://www.envato.com

References

Biszkup, F., Guoth, J., and Horn, P. 1976. Haszongalamb-tenyésztés. Pages 5-11 in: A haszongalambtenyésztés jelentősége. Pages 55-92 in: Haszongalambfajták. Biszkup F., Horn P. (eds) Budapest, Hungary.

Casanova, P. M. P. 2013. Morphological similarities between Spanish pigeon breeds. Turkish J. Vet. Anim. 37:346-351. doi:10.3906/vet-1111-22

Darwin, C. 1859. Variation under domestication. Pages: 7-43. in: On the origin of species by means of natural selection, or the preservation of favoured races in the struggle for life. John Murray, London.

Driscoll, C. A., Macdonald, D. W., and O'Brien, S. J. 2009. From wild animals to domestic pets, an evolutionary view of domestication. PNAS. 106:9971-9978. doi: 10.1073/pnas.0901586106.

Eggleston, E. H. 1921. American Squab Culture. Second Edition, American Pigeon Journal Company Warrenton, MO., U. S. A. 1-278.

Fülöp, L. 2023. A magyar óriás galamb kialakulása. Magyar Óriás Galambtenyésztők Fajtaklub, Klubhíradó. 2-4.

He, W. X., and Jia, J. F. 2015. The complete mitochondrial genome of the Jacobin pigeon (Columba livia breed Jacobin). Mitochondrial DNA, 26: 493-494. doi: 10.3109/19401736.2015.1007322

Horn, P. 1991. Galambtenyésztők kézikönyve. Mezőgazdasági Kiadó, Budapest, Hungary. 1-460.

Kabir, M. A. 2015. Most Beautiful Pigeons of Bangladesh. Vet. Anim. Sci. 3:34-37. doi: 10.11648/j.cb.20150302.12

Mackrott H. 2012. Galambtenyésztés. Mezőgazda Kiadó, Budapest, Hungary. 1-165.

Pacheco, G., Van Grouw, H., Shapiro, M. D., Gilbert, M. T. P., and Vieira, F. G. 2020. Darwin's fancy revised: an updated understanding of the genomic constitution of pigeon breeds. GBE. 12:136-150. doi: 10.1093/gbe/evaa027.

Sell, A. 2012. Pigeon Genetics Applied Genetics in the Domestic Pigeon. Verlag Sell publishing, Germany. 1-528.

Shapiro, M. D., and Domyan, E. T. 2013. Domestic pigeons. Curr. Biol. 23:1-5. doi: 10.1016/j.cub.2013.01.063.

Szűcs, L., and Szécsényi, I. 1965. Galambtenyésztés. Pages: 10-12 in A házigalamb származása. Pages 12-16 in A galambtartás és tenyésztés története. Szűcs, L., (ed) Budapest, Hungary.
Dissel, T. 2018. The Bokhara Trumpeter. This is a publication by the online magazine www.aviculture-europe.nl ISSN: 2352-2445 1-16.

Vickrey, A. I., Domyan, E. T., Horvath, M. P., and Shapiro, M. D. 2015. Convergent Evolution of Head Crests in Two Domesticated Columbids Is Associated with Different Missense Mutations in EphB2. Mol. Biol. Evol. 32:2657-2664. doi: 10.1093/molbev/msv140.

Winkler, J. 1925. Galambtenyésztés. Budapest, Hungary. 1-155.

Source from the Internet:
https://eibel.hu/king-avagy-king-galamb
