# Supplementary material for: MtDNA genetic diversity and phylogeographic insights into giant domestic pigeon (Columba livia domestica) breeds: connections between Central Europe and the Middle East
Source: Poult Sci. 2024 Sep 7;103(12):104310. doi: 10.1016/j.psj.2024.104310 (PMC11458985; doi:10.1016/j.psj.2024.104310)
Supplement: Supplementary file 3 [file mmc3.pdf]

PIGEON DOMESTICATION: A PHYLOGEOGRAPHIC STUDY

MtDNA Genetic Diversity and Phylogeographic Insights into Giant Domestic Pigeon (*Columba livia domestica*) Breeds: Connections Between Central Europe and the Middle East

K. Balog, A. S. Wadday, B. A. Al-Hasan, G. Wanjala, Sz. Kusza, P. Fehér, V. Stéger, Z. Bagi[1]

Supplementary File 3: Genetic diversity indices based on the breeds studied

Indices / Grouping	Number of elements (n)	Number of polymorphisms	Number of haplotypes	Haplotypes in each breed	Haplotype diversity (H_d) ± SD	Nucleotide diversity (π) ± SD
**Bokhara Trumpeter**	7	1	2	Hap_2; Hap_19	0.286+/- 0.196	0.001 +/- 0.002
**Blue Sovater**	11	18	2	Hap_2; Hap_14;	0.182+/- 0.144	0.016+/- 0.001
**Buga pigeon**	12	3	3	Hap_2; Hap_10; Hap_24	0.318+/-0.164	0.002+/- 0.003
**Carnao**	10	1	2	Hap_2; Hap_24	0.356+/-0.159	0.002+/- 0.002
**Hubbel**	10	2	3	Hap_2; Hap_4; Hap_24	0.378+/-0.181	0.002+/- 0.002
**Hungarian Chicken pigeon**	8	0	1	Hap_2	0.000+/- 0.000	0.000 +/- 0.000
**Hungarian Cropper**	14	2	3	Hap_2; Hap_16; Hap_24	0.275+/-0.148	0.001+/- 0.002
**Hungarian Domestic pigeon**	9	3	2	Hap_2; Hap_26	0.222+/-0.166	0.003+/- 0.003
**Hungarian Giant pigeon**	15	42	5	Hap_2; Hap_9; Hap_21;	0.629+/-0.125	0.028+/- 0.016

				Hap_23; Hap_24		
**Hungarian Peasant pigeon**	10	61	2	Hap_1; Hap_2	0.200+/-0.154	0.059+/- 0.033
**Iraqi Raabi pigeon**	50	2	3	Hap_2; Hap_13; Hap_24	0.191+/-0.074	0.001+/- 0.001
**Iraqi Red pigeon**	47	4	4	Hap_2; Hap_17; Hap_18; Hap_25	0.125+/-0.065	0.001+/- 0.001
**Jacobin**	10	120	6	Hap_7; Hap_11; Hap_12; Hap_15; Hap_22; Hap_27	0.778+/-0.137	0.189+/- 0.102
**King**	11	1	2	Hap_2; Hap_4	0.182+/-0.144	0.001+/- 0.001
**Mirthys**	10	34	3	Hap_2; Hap_8; Hap_24	0.378+/-0.181	0.033+/- 0.019
**Mondain**	10	32	4	Hap_2; Hap_4; Hap_5; Hap_12	0.533+/-0.180	0.032+/- 0.019
**Salonta Giant**	13	66	5	Hap_2; Hap_3; Hap_6; Hap_19; Hap_20;	0.731+/-0.096	0.052+/- 0.029
**Runt pigeon**	10	1	2	Hap_2; Hap_24	0.356+/-0.159	0.002+/- 0.002
**Texan**	9	0	1	Hap_2	0.000+/-0.000	0.000 +/- 0.000
