# Supplementary material for: MtDNA genetic diversity and phylogeographic insights into giant domestic pigeon (Columba livia domestica) breeds: connections between Central Europe and the Middle East
Source: Poult Sci. 2024 Sep 7;103(12):104310. doi: 10.1016/j.psj.2024.104310 (PMC11458985; doi:10.1016/j.psj.2024.104310)
Supplement: Supplementary file 4 [file mmc4.pdf]

PIGEON DOMESTICATION: A PHYLOGEOGRAPHIC STUDY

MtDNA Genetic Diversity and Phylogeographic Insights into Giant Domestic Pigeon (*Columba livia domestica*) Breeds: Connections Between Central Europe and the Middle East

K. Balog, A. S. Wadday, B. A. Al-Hasan, G. Wanjala, Sz. Kusza, P. Fehér, V. Stéger, Z. Bagi[1]

Supplementary File 4 Pairwise Fst values heat maps based on the origin of the breeds

Breeds	American pigeons	Asian pigeons	Breeds from the Great Hungarian Plain	Hungarian squab pigeons	Mediterranean pigeons
American pigeons					
Asian pigeons	0.0093				
Breeds from the Great Hungarian Plain	0.001	0.0033			
Hungarian squab pigeons	0.022	0.0033	-0.0011		
Mediterranean pigeons	0.0007	0.0083	-0.0013	0.0073	

The values marked in green represent high values, and the low values are marked in red.

[1] Correspondence should be addressed to Zoltán Bagi, Centre for Agricultural Genomics and Biotechnology, University of Debrecen, 4032, Debrecen, Hungary, Tel:
+36 52 508 444 / 88521, 68304, Email: bagiz@agr.unideb.hu
