# Supplementary material for: MtDNA genetic diversity and phylogeographic insights into giant domestic pigeon (Columba livia domestica) breeds: connections between Central Europe and the Middle East
Source: Poult Sci. 2024 Sep 7;103(12):104310. doi: 10.1016/j.psj.2024.104310 (PMC11458985; doi:10.1016/j.psj.2024.104310)
Supplement: Supplementary file 5 [file mmc5.pdf]

PIGEON DOMESTICATION: A PHYLOGEOGRAPHIC STUDY

MtDNA Genetic Diversity and Phylogeographic Insights into Giant Domestic Pigeon (*Columba livia domestica*) Breeds: Connections Between Central Europe and the Middle East

K. Balog, A. S. Wadday, B. A. Al-Hasan, G. Wanjala, Sz. Kusza, P. Fehér, V. Stéger, Z. Bagi[1]

Supplementary File 5. Pairwise Fst values heat maps based on the levels of the breeds studied

Breeds	Bokhara Trumpeter	Blue Sovater	Buga pigeon	Carnao	Hubbel	Hungarian Chicken pigeon	Hungarian Cropper	Hungarian Domestic pigeon	Hungarian Giant pigeon	Hungarian Peasant pigeon	Iraqi Raabi pigeon	Iraqi Red pigeon	Jacobin	King	Mirthys	Mondain	Salonta Giant	Runt pigeon	Texan
Bokhara Trumpeter																			
Blue Sovater	-0.0398*																		
Buga pigeon	-0.0162	0.0060*																	
Carnao	0.0592*	0.0044*	-0.0293																
Hubbel	-0.0074	-0.0072*	-0.0401	-0.0494*															
Hungarian Chicken pigeon	0.0204	-0.0317*	-0.0373	0.0805	-0.0242														
Hungarian Cropper	0.0000*	0.0148*	-0.0283	-0.017*	-0.0393	-0.0452													
Hungarian Domestic pigeon	-0.0130	-0.0133*	0.0043	0.0457	-0.0185	-0.0141	0.0181												
Hungarian Giant pigeon	-0.0413	0.0050*	-0.0114	-0.0343	-0.0251	-0.0323	0.0016*	-0.0183											
Hungarian Peasant pigeon	-0.0380	0.0057*	0.0147	0.0035*	-0.0032	-0.0242	0.0333	-0.0135	-0.0321										
Iraqi Raabi pigeon	0.0763	0.1492*	0.0330	0.0971*	0.0257	-0.0562	-0.0034	0.1184	0.1102	0.1981									
Iraqi Red pigeon	0.0247	0.1262*	0.0363	0.1098	0.0340	-0.0591	0.0123	0.0725	0.1096*	0.1801	0.0109								
Jacobin	0.4185	0.4700*	0.5045	0.4754*	0.4748	0.4408	0.5326	0.4562	0.4531	0.3462	0.7592	0.7470							
King	0.0128	0.0000*	-0.0036	0.0803*	-0.0639	-0.0317	-0.0044	-0.0126	-0.0043	0.0067	0.0235	-0.0005	-0.0639						
Mirthys	-0.0368	0.0034*	0.0090	-0.0050*	-0.0056	-0.0242	0.0274	-0.0093	-0.0054	-0.0117	0.1864	0.1694	0.4377	0.0095					
Mondain	0.1058	0.1167*	0.1714	0.1572*	0.1173	0.1267	0.2007*	0.1164	0.0472	-0.0105	0.4420	0.4126	0.3795	0.1368	0.0840				
Salonta Giant	-0.0229	0.0420*	0.0524	0.0394*	0.0373	0.0152	0.0703	0.0284	0.0124	-0.0148	0.2360	0.2197	0.4002	0.0480	0.0108	0.0539			
Runt pigeon	0.0592	0.0044*	-0.0293	-0.111*	-0.0494	0.0805*	-0.0172	0.0457*	-0.0343	0.0035	0.0971	0.1098*	0.4754	0.0803	-0.0050	0.1572	0.0394		
Texan	0.0382*	-0.0196*	-0.0259	0.0966	-0.0112	0.0000	-0.0347*	0.0000*	-0.0217	-0.0112	-0.0481*	-0.0510*	0.4599*	-0.0196*	-0.0112	0.1440*	0.0278*	0.0966	

The asterisks mark the statistically significant values (p<0.05), the values marked with green represent high values, and the low values are marked with red

[1] Correspondence should be addressed to Zoltán Bagi, Centre for Agricultural Genomics and Biotechnology, University of Debrecen, 4032, Debrecen, Hungary, Tel: +36 52 508 444 / 88521, 68304, Email: bagiz@agr.unideb.hu
