# Supplementary material for: MtDNA genetic diversity and phylogeographic insights into giant domestic pigeon (Columba livia domestica) breeds: connections between Central Europe and the Middle East
Source: Poult Sci. 2024 Sep 7;103(12):104310. doi: 10.1016/j.psj.2024.104310 (PMC11458985; doi:10.1016/j.psj.2024.104310)
Supplement: Supplementary file 8 [file mmc8.pdf]

## PIGEON DOMESTICATION: A PHYLOGEOGRAPHIC STUDY

# MtDNA Genetic Diversity and Phylogeographic Insights into Giant Domestic Pigeon (*Columba livia domestica*) Breeds: Connections Between Central Europe and the Middle East

K. Balog, A. S. Wadday, B. A. Al-Hasan, G. Wanjala, Sz. Kusza, P. Fehér, V. Stéger, Z. Bagi[1]

**Supplementary File 8** The haplotype relationships by the origin of the breeds

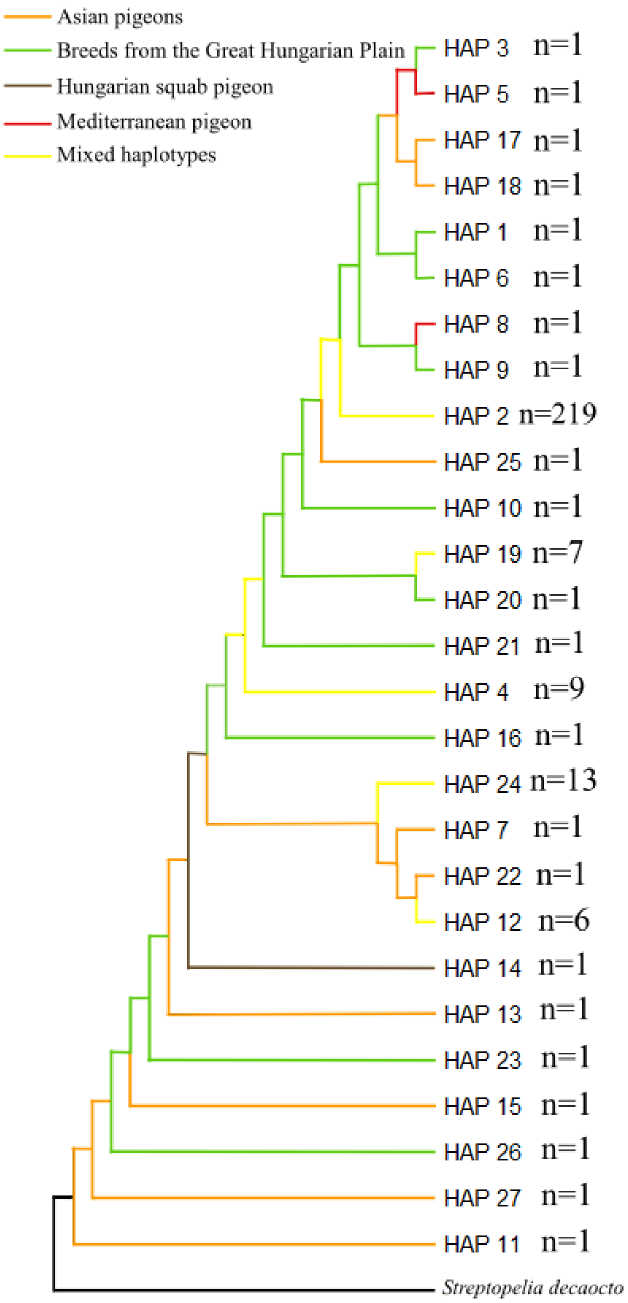


[1] Correspondence should be addressed to Zoltán Bagi, Centre for Agricultural Genomics and Biotechnology, University of Debrecen, 4032, Debrecen, Hungary, Tel:
+36 52 508 444 / 88521, 68304, Email: bagiz@agr.unideb.hu
